# Infiltrating T-cell abundance combined with EMT-related gene expression as a prognostic factor of colon cancer

**DOI:** 10.1080/21655979.2021.1939618

**Published:** 2021-06-27

**Authors:** Xiaowei Huang, Chan Chen, Yajing Xu, Lanxiao Shen, Yi Chen, Huafang Su

**Affiliations:** a Department of Radiation Oncology, First Affiliated Hospital of Wenzhou Medical University, Wenzhou, China; b Department of Geriatric Medicine, First Affiliated Hospital of Wenzhou Medical University, Wenzhou, China; c Department of Radiotherapy Center, First Affiliated Hospital of Wenzhou Medical University, Wenzhou, China; d Department of Oncology-Pathology, Karolinska Institutet, Sweden

**Keywords:** Colon cancer, EMT, T-cells, immune checkpoint blockade, survival

## Abstract

EMT-related gene expression reportedly exhibits correlation with the anti-tumor immunity of T cells. In the present study, we explored the factors that might affect the efficacy of immunotherapy in colon cancer with treatment. In this regard, RNA-seq and clinical data of 469 colon cancer samples derived from the Cancer Genome Atlas (TCGA) database were used to calculate infiltrating T-cell abundance (ITA), to illustrate a pathway enrichment analysis, and to construct Cox proportional hazards (CPH) regression models. Subsequently, the RNA-seq and clinical data of 177 colon cancer samples derived from the GSE17536 cohort were used to validate the CPH regression models. We found that ITA showed correlation with EMT-related gene expression, and that it was not an independent prognostic factor for colon cancer. However, upon comparison of two groups with the same ITA, higher EMT expression helped predicted a worse prognosis, whereas a higher ITA could help predict a better prognosis upon comparison of two groups with the same EMT. Additionally, seven genes were found to be statistically related to the prognosis of patients with colon cancer. These results suggest that the balance between ITA and EMT-related gene expression is conducive to the prognosis of patients with colon cancer, and TPM1 is necessary to further explore the common target genes of immune checkpoint blockade.

## Graphical Abstract

**Figure:**
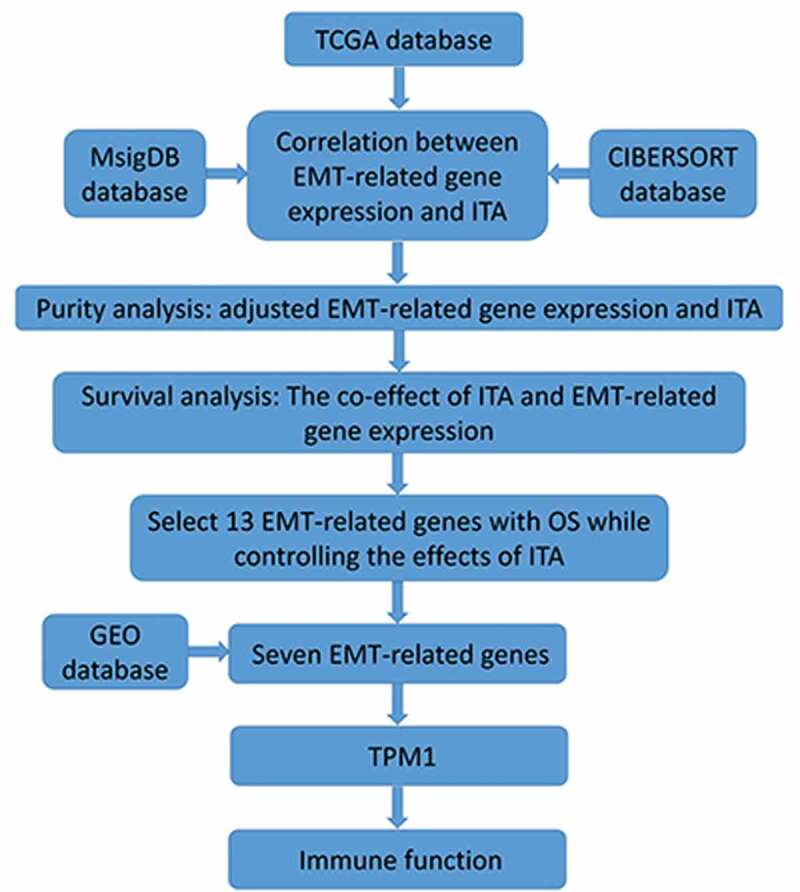

## Introduction

In clinical practice, colon cancer is a commonly reported malignant tumor that affects the digestive system. The global cancer statistics for 2020 showed that the incidences and mortalities associated with colon cancer were ranked third and second, respectively [1]. Surgery is the primary colon cancer treatment intervention, however, the five-year survival rate of patients who undergo surgery for colon cancer is not considerable. Approximately 61%, 57%, and 32% of the patients were reportedly alive after five years of opting for a surgical intervention in North America, Japan, and China, respectively [2]. Notably, even if the residual tumor cells are subjected to routine postoperative radiotherapy and chemotherapy, their elimination is difficult [3,4]. Therefore, it is crucial to explore new treatments to improve the prognosis of patients with colon cancer.

Immune checkpoint blockade (ICB) therapy is an attractive option, but its therapeutic effect on colon cancer is unsatisfactory [5]. ICB improves the anti-tumor response of the immune system by regulating T-cell activity. The inhibitors that are commonly used in clinical practice include monoclonal antibodies against programmed death receptor 1 (PD-1), programmed death receptor-ligand 1 (PD-L1), and cytotoxic T lymphocyte-associated protein 4 (CTLA-4) [6]. Reactivated T-cells can perform the recognition and elimination of tumor cells [7]. Studies have indicated that high T‐cell infiltration is correlated with a favorable prognosis in colon cancer [8,9]. Thus far, the factors influencing T-cell anti-tumor effects have not been comprehensively researched. The present study aims to highlight new perspectives for improving the prognosis of patients with colon cancer.

Studies have confirmed the possible inhibition of the anti-tumor response of the immune system by epithelial-mesenchymal transition (EMT)- related gene expression in lung cancer and liver cancer patients through the inhibition of CD8^+^ cytotoxic T-cell proliferation [10,11]. However, a few studies have concluded that tumors with high EMT scores, such as melanoma, kidney cancer, and bladder cancer, exhibit better efficacy with CTLA-4, PD-1, or PD-L1 antibodies [12]. As such, whether T-cells anti-tumor effects on colon cancer are correlated with EMT-related gene expression is rarely reported.

In summary, we hypothesized that infiltrating T-cell abundance combined with EMT-related gene expression could be considered a prognostic factor for colon cancer. To improve the prognosis of immunotherapy patients with colon cancer, we explored the mechanism of EMT-related gene expression in the anti-tumor process of T cells. By referencing transcriptome data and clinical information collected from patients with colon cancer from TCGA and CIBERSORT databases, along with the Molecular Signatures Database (MsigDB), we analyzed correlation, pathway enrichment, tumor purity, and overall survival (OS) of patients with colon cancer. Subsequently, we utilized data for colon cancer samples from the GSE17536 cohort to validate the CPH regression models. The findings led to an inference that the balance between ITA and EMT-related gene expression correlated with improved overall patient survival in colon cancer cases. Additionally, seven genes were statistically related to prognosis in patients with colon cancer, and TPM1 provided a theoretical basis for co-targeting ICB and EMT-related gene expression.

## Materials and methods

### Data sources

The gene signature feature set of 22 different types of immune cells in varying states was retrieved from the CIBERSORT database (https://cibersort.stanford.edu/) [13]. We retrieved the hallmark gene sets from the MsigDB database (http://www.gsea-msigdb.org/gsea/msigdb/index.jsp) [14]. The clinical data and RNA sequencing data of the patients with colon cancer were retrieved from TCGA database (https://confluence.broadinstitute.org/display/GDAC/Home/), and our final dataset consisted of 469 cases consisting of both clinical information and corresponding RNA-seq data. Data on the GSE17536 cohort containing 177 colon cancer samples were retrieved from the Gene Expression Omnibus (GEO) database (https://www.ncbi.nlm.nih.gov/geo/query/acc.cgi) [15].

### Infiltrating T-cell abundance (ITA) calculation method

The gene expression of immune cell markers has been extensively considered to evaluate the abundance of tumor-infiltrating T cells and that of various other types of blood cells [16–19]. This method was adopted to estimate the infiltrating T-cell abundance (ITA) in TCGA colon cancer cohort. The application function in R (version: 4.0.3 https://cran.r-project.org/bin /windows/base/) was used for expression value normalization for gene profiles of 22 types and states of immune cells. Genes with an expression value for T cells greater than 2 were considered T-cell marker genes, while ITA was defined as the arithmetic mean of each colon cancer sample transformed into a logarithmic T-cell marker gene expression profile.

### Enrichment and correlation analysis

Gene set enrichment analysis of each colon cancer case was conducted using functions in the Bioconductor library ‘GSVA’ (version 1.36.3 https://www.bioconductor.org/packages/release/bioc/html/GSVA.html) [14,20]. The cor() function in R was used to analyze the correlations between ITA and the individual gene set corr. The test () function was used to calculate the corresponding P value.

### EMT-related gene expression

The expression signature of EMT-related genes consisted of 200 genes whose data were obtained from the hallmark gene set. Using each TCGA database sample, we calculated an EMT-related gene expression score using the arithmetic mean of 200 EMT-related genes (log2 scale).

### Purity analysis

Tumor purity analysis was performed to study aspects of the tumor microenvironment [16,21]. The ESTIMATE algorithm was used to generate two scores (stromal and immune scores), which were used to deduce the immune and stromal cell component ratios in tumor samples and to combine them to evaluate tumor purity [16]. The tumor purity of colon cancer cases was analyzed using functions in the R library ‘estimate’ (version 1.0.13 https://bioinformatics.mdanderson.org/estimate/rpackage.html) [21]. After performing negative logarithmization, further analysis was conducted to determine whether the tumor purity ITA and EMT-related gene expression were correlated using the following linear regression model: ITA~1+ log(1-purity). The model’s residual value was obtained and considered as the adjusted ITA. EMT-related gene expression values were adjusted using the same method.

### Survival analysis

The Cox Proportional Hazards (CPH) regression model was used for assessing the dependence of overall survival (OS) on ITA and EMT-related gene expression levels for several patients with colon cancer using the R library ‘survival’ (version 3.2–7 https://cran.r-project.org/web/packages/survival/index.html) [22]. Hazard ratios (HRs) were used to summarize the extent of the associations, while reported HRs were scaled in the comparison of biomarker scores at the 75th and 25th percentiles. The Wald test was used as the basis for obtaining two-sided 95% confidence intervals for the HRs. Additionally, we assessed association with OS for each EMT-related gene while controlling for the effects of ITA. Then, the EMT-related genes were categorized based on P-value according to the Wald test results. We used a cutoff of p < 0.05 to select 13 EMT-related genes. Subsequently, these genes were tested for prognostic significance in the GSE17536 cohort.

### Comprehensive analysis

The Human Protein Atlas (HPA) database (https://www.proteinatlas.org) was used to analyze the protein expression. The HPA provides data on 48 colon cancer tissue samples and 6 normal tissue samples, and provides downloadable protein expression images and gene expression data for immune cells [23]. TISIDB is used to pre-calculate the association between any gene and immune function (such as immunoimmunomodulation and chemokine secretion) in various cancers [24]. We determined the correlation between our target gene and immune function.

## Results

### The combined effect of EMT-related gene expression and infiltrating T cells on the prognosis of patients with colon cancer

Inhibition of the anti-tumor response of the immune system by epithelial-mesenchymal transition (EMT)- related gene expression in lung cancer and liver cancer has been previously reported [10,11]. We studied the correlation between EMT-related gene expression and infiltrating T-cell abundance and performed a survival analysis. Consistent with the findings reported in previous studies, we found that the expression of EMT-related genes inhibited the anti-cancer effect of T cells. Therefore, we performed screening for seven genes using the GEO dataset and the expression of EMT-related genes and protein levels. These genes are highly correlated with colon cancer prognosis. The protein expression levels of the indicated seven genes were compared, and it was found that TPM1 was statistically different between the tumor and normal groups; subsequently, we explored the relationship between TPM1 and immune function.

### Correlation between EMT-related gene expression and ITA

**Figure 1.**
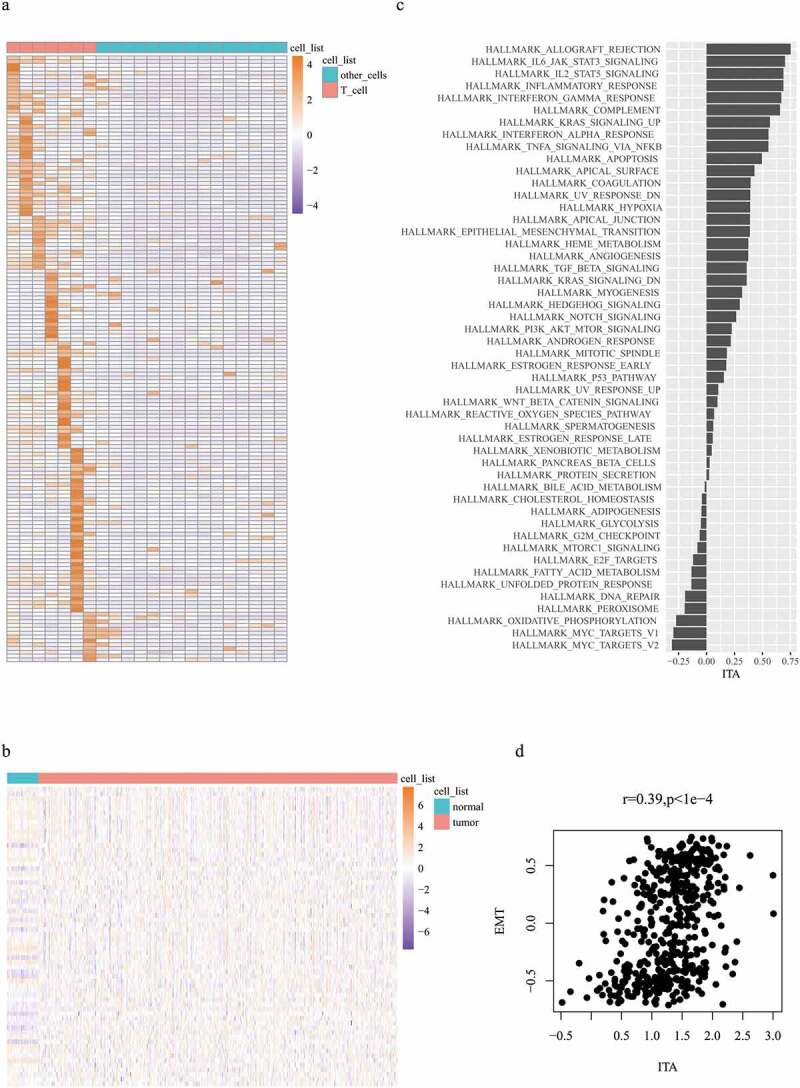
Expression profiles of 159 T-cell-labeled genes within 22 different immune cell subtypes (a); expression profiles of 153 T-cell-labeled genes across colon cancer cohort (b); ITA correlation comparison with 50 pathways (c); plot illustrating the correlation between EMT and ITA (d)

Differentially expressed genes between T cells and other immune cells were estimated to evaluate the abundance of tumor-infiltrating T cells. A total of 159 significant genes were selected as T-cell marker genes (Figure 1a). The expression of these genes in colon cancer samples represents ITA. Here, 153 T-cell marker genes were expressed in normal samples and colon cancer samples (Figure 1b). The next step involved the exploration of a gene set whose expression was related to ITA. Data on 50 gene sets were obtained; the highly enriched gene sets that were positively correlated with ITA included EMT, hypoxia, and immune-related pathways, such as inflammatory, interferon, and tumor necrosis factor (Figure 1c). Subsequently, we focused on the examination of the relationship between EMT-related gene expression and ITA. EMT-related gene expression was positively correlated with ITA (r = 0.39, p < 1e-4; Figure 1d).

### Correlation between EMT-related gene expression and ITA adjusted by purity analysis

**Figure 2.**
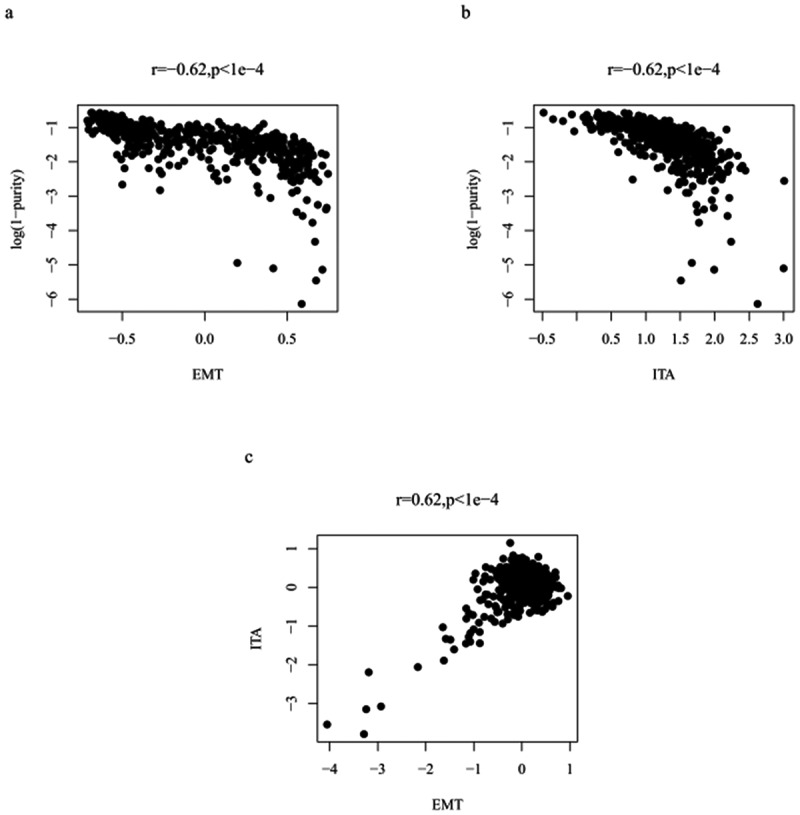
Plot illustrating the correlation between adjusted EMT and tumor purity (a); plot depicting the correlation between adjusted ITA and tumor purity (b); plot highlighting the adjusted EMT and ITA correlation (c)

To distinguish between EMT genes expressed by tumor cells or stromal cells, we calculated the tumor purity of each sample. We then assessed the correlation between EMT-related gene expression and tumor purity to distinguish between EMT-related gene expression, mainly related to tumor cells or non-tumor cells. They were positively correlated (r = −.062, p < 1e-4; Figure 2a). Therefore, the expression values of EMT-related genes were adjusted (Table. S1). We also assessed the correlation between ITA and tumor purity (r = −.062, p < 1e-4; Figure 2b), and adjusted the ITA value (Table. S2). Finally, the EMT-related gene expression and ITA correlation in colon cancer cases showed improvements after purity analysis (r = 0.62, p < 1e-4; Figure 2c).

### The disparate impact of EMT-related gene expression and ITA on survival among the patients in the colon cancer cohort

**Figure 3.**
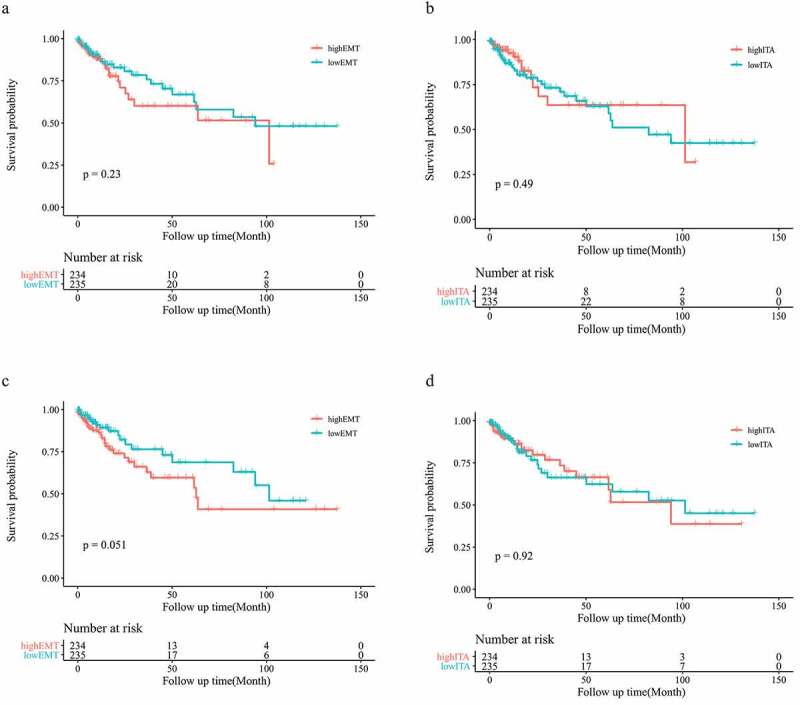
Survival analysis of the original EMT grouping (a); survival analysis of the original ITA grouping (b); survival analysis of the adjusted EMT grouping (c); survival analysis of the adjusted ITA grouping (d)

To explore the effect of upregulation of EMT-related gene expression in patients with colon cancer, we analyzed the prognosis of patients. No statistically significant differences were observed between the low- and high-EMT groups in OS (p = 0.23) (Figure 3a). We also analyzed the influence of the number of infiltrating T cells on patient prognosis. The low- and high-ITA groups showed no statistically significant differences (p = 0.49; Figure 3b). To reduce the influence of stromal cells on the results, we repeated the experimental process using the adjusted data. After conducting purity analysis and adjustment, EMT-related gene expression and ITA were not found to be significantly associated with OS in patients with colon cancer (p = 0.051, p = 0.92; Figure 3(c,d)).

### The effect of ITA combined with EMT-related gene expression on the prognosis of patients with colon cancer patients

**Figure 4.**
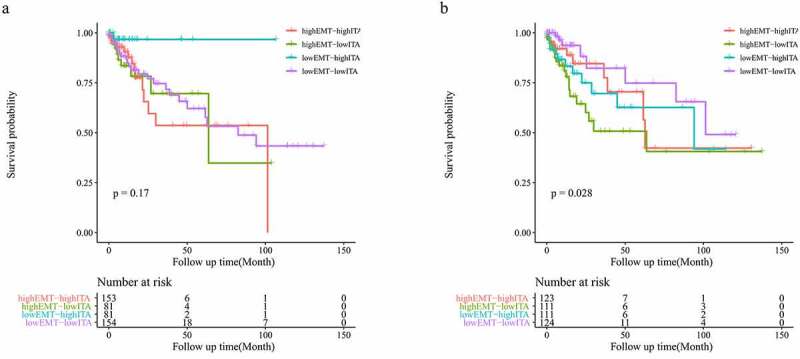
Survival analysis of the original EMT-ITA (a); survival analysis of the adjusted EMT-ITA (b)

Considering the positive correlation observed between ITA and EMT-related gene expression, we aimed to obtain further prognostic and biological insights by combining these parameters. Although no statistically significant differences were identified across the four groups for overall survival when using the original ITA and EMT data (p = 0.17; Figure 4a), the prognosis of the four groups was significantly different based on the adjusted data (p = 0.028; Figure 4b). After conducting purity analysis and adjustment, when comparing two groups with the same EMT-related gene expression, a higher ITA helped predict a better prognosis in patients with colon cancer (Figure 4b). Upon comparing the two groups with the same ITA, the higher expression of EMT-related genes significantly predicted a worse outcome for patients with colon cancer (Figure 4b). Interestingly, patients with high EMT and low ITA presented with the worst prognosis (Figure 4b).

### 13 EMT-related genes associated with prognosis of patients with colon cancer

**Figure 5.**
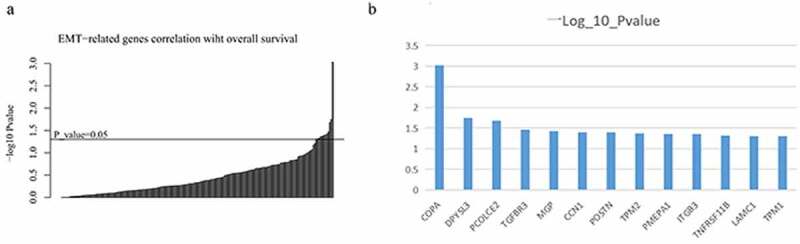
Individual EMT-related genes were categorized based on the significance of their association with survival (a); the Y-axis shows the −log10 P-value estimated by using the Wald test for 13 EMT-related genes in the CPH model (p < 0.05) (b)

To explore target genes that might affect outcomes in patients with colon cancer, we investigated the role of 200 EMT-related genes in their prognosis. The significance of their association with survival was used to classify the individual EMT-related genes (Figure 5a). We obtained data on 13 EMT-related genes that were significantly associated with survival in patients with colon cancer (p < 0.05; Figure 5b).

### GSE17536 cohort validation of seven EMT-related genes associated with prognosis of patients with colon cancer

**Figure 6.**
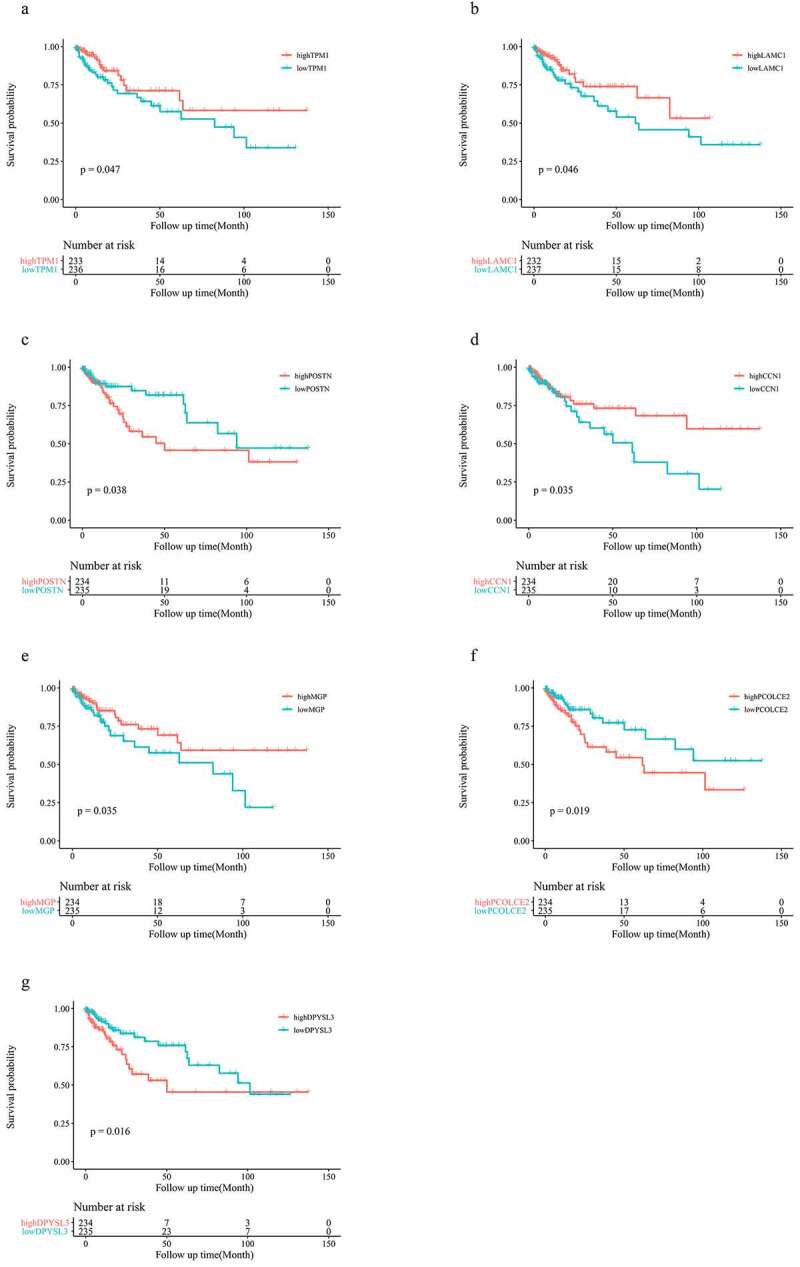
Seven genes are associated with prognosis in the GSE17536 cohort (a-g)

To test the general effect of these 13 genes on prognosis of patients with colon cancer, we utilized information derived from other databases to investigate the prognostic role of these genes in patients with colon cancer. Seven EMT-related genes (TPM1, LAMC1, POSTN, CCN1, MGP, PCOLCE2, and DPYSL3) were associated with OS in patients in the GSE17536 cohort, among which POSTN, PCOLCE2, and DPYSL3 were found to be risk factors, while others played roles as favorable factors (p < 0.05; Figure 6(a-g)).

### Immunohistochemistry analysis and further analyze the correlation between the TPM1 and immunity

**Figure 7.**
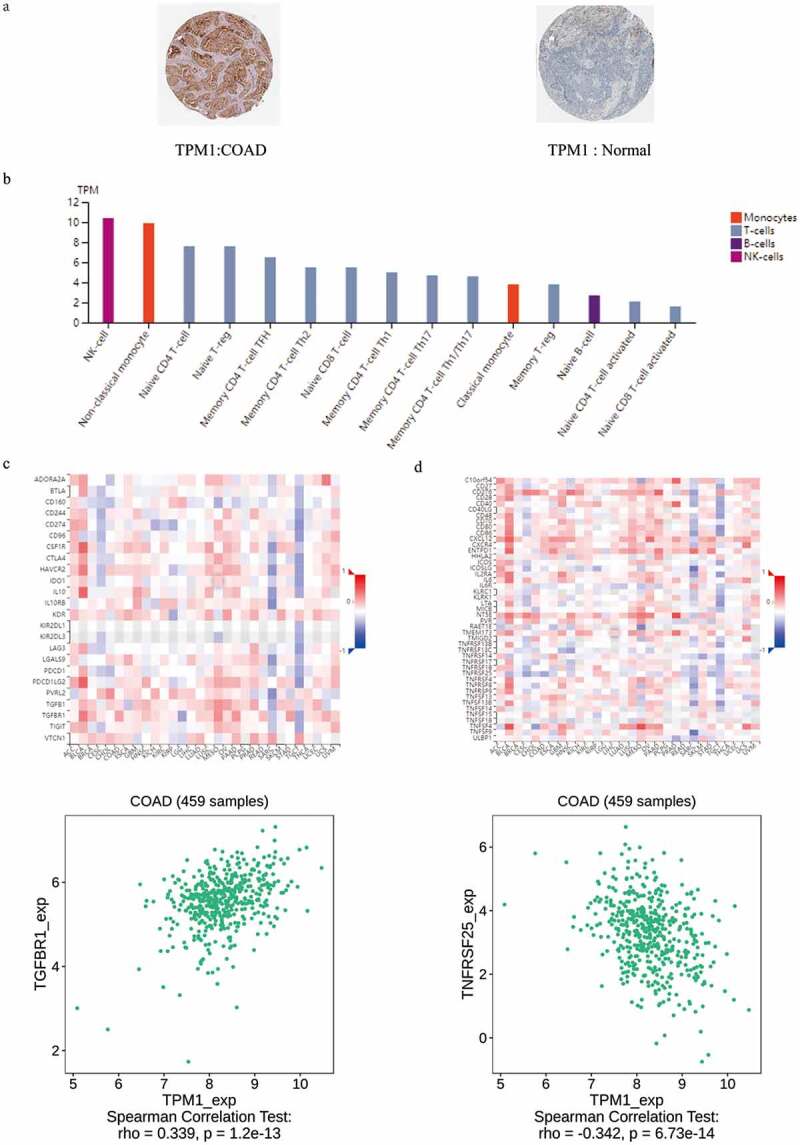
TPM1 expressed in colon cancer and normal (a); TPM1 expressed in different types and stages of immune cells (b); correlation coefficients between expression of TPM1 and immunomodulators (indicated on the Y axis) across various types of human cancers (indicated on the X axis; c); correlation coefficients between expression of TPM1 and immunomodulators (indicated on the Y axis) across various types of human cancers (indicated on the X axis; d)

The immunohistochemistry analysis data derived from HPA showed that in colon cancer tissues, TPM1 expression was lower than that in normal tissues (Figure 7a), and differences in the other six protein expression were statistically insignificant (Table. S3). To explore the mechanism by which TPM1 affected the immune function of T cells, we compared the expression of TPM1 in different types and stages of immune cells, and compared the relationship between TPM1 and immunomodulators. In the T lymphocyte family, TPM1 is mainly expressed in NK cells, non-classical monocytes, naive CD4 + T cells, native T-reg cells, memory CD4 T-cell TFH, and memory CD4 T-cell Th2 cells (Figure 7b). We found that the immunomodulator TGFBR1 showed positive correlation with TPM1 expression in colon cancer (Figure 7c), and the immunomodulator TNFRSF25 showed negative correlation with TPM1 (Figure 7d).

## Discussion

We demonstrated that ITA was positively correlated with tumor purity and was not correlated with colon cancer prognosis. Our results indicate that the T-cell immune system anti-tumor response in patients with colon cancer is inhibited. CD8 ^+^ cells play a crucial role in the elimination of tumor cells in T-cell types and states [25]. The anti-tumor immune effect of CD8^+^ T cells can be enhanced by the action of CD4^+^ T cells, while Treg cells inhibit CD8^+^ T-cell proliferation [25,26]. However, tumor cells can induce a significant increase in the number of Treg cells to escape recognition and elimination by CD8^+^ T cells [27,28].

ICB was then developed to block Treg cell-mediated immunosuppression [29]. It exerts a poor therapeutic effect on patients with colon cancer [30]. Therefore, exploration of the factors that influence the anti-tumor effects of ICB is warranted. We found that EMT-related gene expression might inhibit the immune system’s anti-tumor response in T cells. We then screened out the top seven EMT-related genes, namely TPM1, LAMC1, PCOLCE2, POSTN, CCN1, MGP, and DPYSL3. We found that TPM1 was positively related to certain immune cells and was positively related to a few immune regulatory genes.

Unfortunately, ITA showed positive correlation with EMT-related gene expression in bladder cancer, breast cancer, lung cancer, and metastatic melanoma [10,31–34], and was confirmed in colon cancer in our study. The number of T cells is not affected; however, the function may be suppressed. This result suggests that to improve the prognosis of patients with colon cancer, there is a need to further investigate ITA and EMT-related gene expression.

Biologically, epithelial cells are altered during EMT for the formation of mesenchymal cells [35]. However, in tumor pathology, cancer cells often undergo partial or incomplete EMT [36]. This aspect is crucial for formulating detection methods to identify EMT programs in cancer cells [37]. Therefore, we used EMT-related gene expression to identify EMT in colon cancer. However, both cancer and stromal cells express EMT-related genes [38]. We further demonstrated that EMT-related gene expression correlated with tumor purity in colon cancer. This result confirmed that EMT was mainly expressed in colon cancer cells rather than stromal cells. Therefore, it can be concluded that colon cancer cells undergo EMT.

To investigate the role of EMT in the process of T cells against colon cancer cells, we first compared two groups with the same EMT-related gene expression and found that a higher ITA could help predict a better prognosis in patients with colon cancer. Before categorizing EMT-related genes into high-expression and low-expression groups, ITA does not play a remarkable role in the prognosis of colon cancer; hence, the result obtained after grouping shows that the expression of EMT-related genes is an influencing factor in the anti-tumor process of T cells. Second, we compared two groups with the same ITA and found that higher EMT-related gene expression could help predict a worse prognosis in patients with colon cancer. These results indicate that the expression of EMT-related genes is negatively correlated with the anti-tumor effect of T cells.

As previously discussed, EMT-related gene expression inhibits the function of T cells rather than aiding the reduction in the number of T cells. Research has shown that EMT may trigger tumor promotion by affecting the composition of the tumor microenvironment and immune system infiltration [39]. The key aspect of the effect of EMT on the immune system is that it upregulates the gene expression of the immune checkpoint in lung and breast cancer cells [40–43].

Conversely, EMT has also been shown to induce immunosuppression in cancer [39–44]. EMT-activating transcription factors (EMT-TFs) upregulate pro-inflammatory and immunosuppressive cytokine expression in cancer cells and induce EMT [38,39]. EMT-TFs demonstrate multiple functions in cancer biology, are involved in the repair of double-stranded DNA, escape senescence, and induce pro-survival and anti-apoptotic phenotypes [45–48]. The complex functions of EMT-TFs result in their anti-immune effects, which are likely to be affected by other factors. Studies have established that EMT-TFs, under the action of hypoxia-inducible factor −1α (HIF1A), resisted the anti-tumor effect of CD8 + T cells [40]. We also demonstrated that gene expression related to hypoxia could correlate with ITA in colon cancer cells, supporting the results mentioned in previous studies.

Additionally, the balance of ITA and EMT-related gene expression may have predictive or prognostic implications in patients with colon cancer undergoing treatment with ICB. In our study, the high EMT-low ITA group showed the worst prognosis, while the low EMT-low ITA group demonstrated the best prognosis. This finding may help explain the existence of less number of T cells infiltrating colon cancer. As corroborated by a lung cancer study, EMT-related gene expression considerably influences the prognosis of patients with colon cancer. In this study, significant Th9 and Th17 cell accumulation induced EMT in lung cancer cells and correlated with poor survival in lung cancer patients [49]. Therefore, it is imperative to identify and distinguish the state, type, and number of tumor-infiltrating T cells, which can influence the effect of EMT on the prognosis of patients with colon cancer. While EMT was initially considered a binary process, its description as a dynamic pathway with intermediate states is now well supported [50]. Therefore, it has become increasingly apparent that these intermediate phenotypes should be subjected to quantitative assessments for consideration of novel therapeutic design strategies [51].

Recent reports have highlighted the categorization of EMT-related gene scores from low to high to evaluate the relationship between EMT and the prognosis of patients with different cancers [52]. However, we did not adopt this quantitative scoring system. In our study, EMT-related gene expression was not significantly correlated with the prognosis of patients with colon cancer, but was positively correlated with tumor purity. Furthermore, the results support the notion that EMT-related genes should be subjected to quantitative assessments for consideration of novel therapeutic design strategies.

We identified seven EMT-related genes (TPM1, LAMC1, POSTN, CCN1, MGP, PCOLCE2, and DPYSL3) with prognostic significance in patients with colon cancer from TCGA and GSE17536 cohorts. The high expression of these seven genes was detrimental to the poor prognosis of patients with digestive tumors [53–59]. These genes may be associated with the target genes.

The expression of TPM1 in colon cancer cells was lower than that in normal cells. Studies have shown that TPM1 can increase the sensitivity of colon cancer cells to chemotherapy drugs and can exert an inhibitory effect on colon cancer cells [60], which is consistent with our findings. TPM1 inhibits tumor malignant progression by regulating immune cell proliferation and development. In addition to CD8 + T cells, CD4 + T cells and CD4 T-cell TFH may also demontrate particularly potent antitumor activity [61]. Latest reports state that TGF-βsignals are dispensable for maintaining steady-state Treg cell homeostasis and for Treg cell suppression of T cell proliferation and T helper-1 (Th1) cell differentiation [62], TNFRSF25 can induce a substantial number of T-reg cells to undergo proliferation, and the induced T-reg cells exert immunosuppressive effects [63]

## Conclusion

In conclusion, we identified that the balance between EMT and ITA-related gene expression might have predictive or prognostic implications for patients with colon cancer subjected to treatment with ICB. Moreover, we efficiently identified seven EMT-related genes with prognostic value for colon cancer. Finally, we found that the expression of TPM1 occurred in NK cells, non-classical monocytes, naive CD4 + T cells, native T-reg cells, memory CD4 T-cell TFH, and memory CD4 T-cell Th2, and the findings indicated that TPM1 might possess immunomodulatory functions; therefore, further exploration of the common target genes of ICB and EMT-related gene expression in colon cancer is necessary.

## Supplementary Material

Supplemental Material
