# Supplementary material for: Infiltrating T-cell abundance combined with EMT-related gene expression as a prognostic factor of colon cancer
Source: Bioengineered. 2021 Jun 27;12(1):2688–701. doi: 10.1080/21655979.2021.1939618 (PMC8806648; doi:10.1080/21655979.2021.1939618)
Supplement: Supplemental Material [file KBIE_A_1939618_SM4068.zip › supplementary/Table_S1.docx]

| **Table S1:EMT value adjusted by purity analysis** | | | | |
| --- | --- | --- | --- | --- |
|  | x |  |  |  |
| TCGA.AA.3854.01A.01R.0905.07 | 0.107948031 |  |  |  |
| TCGA.CM.6169.01A.11R.1653.07 | -0.782292764 |  |  |  |
| TCGA.D5.6530.01A.11R.1723.07 | -0.460307171 |  |  |  |
| TCGA.AA.A00F.01A.01R.A002.07 | 0.695433434 |  |  |  |
| TCGA.A6.5659.01A.01R.1653.07 | 0.590276913 |  |  |  |
| TCGA.AA.3494.01A.01R.1410.07 | -0.015134704 |  |  |  |
| TCGA.AA.A029.01A.01R.A00A.07 | 0.30865282 |  |  |  |
| TCGA.AD.6888.01A.11R.1928.07 | 0.296648168 |  |  |  |
| TCGA.DM.A28M.01A.12R.A16W.07 | 0.179275151 |  |  |  |
| TCGA.DM.A1D0.01A.11R.A155.07 | 0.254127074 |  |  |  |
| TCGA.AA.3544.01A.01R.1873.07 | -0.555025007 |  |  |  |
| TCGA.F4.6805.01A.11R.1839.07 | 0.094706107 |  |  |  |
| TCGA.F4.6569.01A.11R.1774.07 | -0.399716888 |  |  |  |
| TCGA.CM.5863.01A.21R.1839.07 | 0.540816991 |  |  |  |
| TCGA.AA.A01S.01A.21R.A083.07 | 0.232970016 |  |  |  |
| TCGA.CM.6168.01A.11R.1653.07 | -0.194803583 |  |  |  |
| TCGA.AD.6548.01A.11R.1839.07 | 0.223011035 |  |  |  |
| TCGA.AA.A00D.01A.01R.A002.07 | -0.802029148 |  |  |  |
| TCGA.CA.5255.01A.11R.1839.07 | 0.26450188 |  |  |  |
| TCGA.A6.4105.01A.02R.1774.07 | 0.178950721 |  |  |  |
| TCGA.D5.6529.01A.11R.1774.07 | -0.286861072 |  |  |  |
| TCGA.AZ.6601.01A.11R.1774.07 | -0.326074662 |  |  |  |
| TCGA.G4.6306.01A.11R.1774.07 | 0.1162872 |  |  |  |
| TCGA.5M.AAT5.01A.21R.A41B.07 | 0.230891171 |  |  |  |
| TCGA.A6.A566.01A.11R.A28H.07 | -1.623219472 |  |  |  |
| TCGA.AY.4070.01A.01R.1113.07 | 0.343452711 |  |  |  |
| TCGA.AA.3522.01A.01R.0821.07 | -0.102617875 |  |  |  |
| TCGA.AA.3663.01A.01R.1723.07 | -0.092134469 |  |  |  |
| TCGA.AA.3971.01A.01R.1022.07 | -0.393706975 |  |  |  |
| TCGA.A6.2672.01A.01R.0826.07 | -0.477627701 |  |  |  |
| TCGA.A6.6780.01A.11R.A278.07 | -0.716154637 |  |  |  |
| TCGA.CM.6162.01A.11R.1653.07 | -1.162771589 |  |  |  |
| TCGA.CK.6747.01A.11R.1839.07 | 0.196140567 |  |  |  |
| TCGA.A6.4107.01A.02R.1410.07 | 0.210473086 |  |  |  |
| TCGA.AA.3675.01A.02R.0905.07 | 0.276579913 |  |  |  |
| TCGA.F4.6703.01A.11R.1839.07 | -2.934382208 |  |  |  |
| TCGA.AA.3877.01A.01R.1022.07 | -0.014297431 |  |  |  |
| TCGA.A6.6782.01A.11R.1839.07 | 0.016767784 |  |  |  |
| TCGA.A6.6653.01A.11R.1774.07 | 0.542385646 |  |  |  |
| TCGA.AA.3860.01A.02R.0905.07 | 0.262346424 |  |  |  |
| TCGA.G4.6293.01A.11R.1723.07 | -0.149544707 |  |  |  |
| TCGA.G4.6298.01A.11R.1723.07 | 0.955698007 |  |  |  |
| TCGA.CM.4746.01A.01R.1410.07 | 0.107225448 |  |  |  |
| TCGA.AD.A5EJ.01A.11R.A28H.07 | 0.423711135 |  |  |  |
| TCGA.DM.A0XF.01A.11R.A155.07 | 0.176059811 |  |  |  |
| TCGA.A6.6648.01A.11R.1774.07 | 0.097192049 |  |  |  |
| TCGA.A6.5656.01A.21R.A278.07 | 0.04403119 |  |  |  |
| TCGA.AA.3685.01A.02R.A32Z.07 | -0.450802251 |  |  |  |
| TCGA.4N.A93T.01A.11R.A37K.07 | 0.122616022 |  |  |  |
| TCGA.DM.A28C.01A.11R.A32Y.07 | 0.225060678 |  |  |  |
| TCGA.CK.5912.01A.11R.1653.07 | 0.092576084 |  |  |  |
| TCGA.AA.3516.01A.02R.0826.07 | 0.010661152 |  |  |  |
| TCGA.AD.6890.01A.11R.1928.07 | 0.307505233 |  |  |  |
| TCGA.A6.2674.01B.04R.A277.07 | -1.583696125 |  |  |  |
| TCGA.F4.6461.01A.11R.1774.07 | 0.458354155 |  |  |  |
| TCGA.AA.3662.01A.01R.1723.07 | -0.206497947 |  |  |  |
| TCGA.A6.2677.01B.02R.A277.07 | -0.340602109 |  |  |  |
| TCGA.AA.3673.01A.01R.0905.07 | 0.342839495 |  |  |  |
| TCGA.AA.A02R.01A.01R.A00A.07 | -0.327787878 |  |  |  |
| TCGA.D5.6535.01A.11R.1723.07 | 0.219332355 |  |  |  |
| TCGA.D5.6537.01A.11R.1723.07 | 0.261908827 |  |  |  |
| TCGA.4T.AA8H.01A.11R.A41B.07 | 0.235649429 |  |  |  |
| TCGA.AA.3994.01A.01R.1113.07 | 0.440904656 |  |  |  |
| TCGA.CK.5916.01A.11R.1653.07 | 0.02677821 |  |  |  |
| TCGA.RU.A8FL.01A.11R.A37K.07 | 0.2703241 |  |  |  |
| TCGA.F4.6460.01A.11R.1774.07 | 0.248537798 |  |  |  |
| TCGA.AA.A00R.01A.01R.A002.07 | -1.008729249 |  |  |  |
| TCGA.AA.3555.01A.01R.0821.07 | 0.543234049 |  |  |  |
| TCGA.CM.5344.01A.21R.1723.07 | 0.762856067 |  |  |  |
| TCGA.F4.6855.01A.11R.1928.07 | 0.272081479 |  |  |  |
| TCGA.G4.6323.01A.11R.1723.07 | -0.180667972 |  |  |  |
| TCGA.A6.6142.01A.11R.1774.07 | 0.42658793 |  |  |  |
| TCGA.AA.3844.01A.01R.1022.07 | -0.203420522 |  |  |  |
| TCGA.G4.6303.01A.11R.1774.07 | 0.467858987 |  |  |  |
| TCGA.DM.A1HB.01A.21R.A180.07 | 0.477158217 |  |  |  |
| TCGA.A6.5659.01B.04R.A277.07 | -0.053772581 |  |  |  |
| TCGA.D5.5539.01A.01R.1653.07 | 0.320636725 |  |  |  |
| TCGA.AA.3538.01A.01R.0821.07 | 0.568682988 |  |  |  |
| TCGA.AA.A03F.01A.11R.A16W.07 | -0.069323335 |  |  |  |
| TCGA.AA.3660.01A.01R.1723.07 | 0.047918405 |  |  |  |
| TCGA.CM.5860.01A.01R.1653.07 | 0.328498467 |  |  |  |
| TCGA.AA.3975.01A.01R.1022.07 | -0.140826512 |  |  |  |
| TCGA.A6.2674.01A.02R.A278.07 | -1.071942918 |  |  |  |
| TCGA.AZ.6607.01A.11R.1839.07 | -0.110909486 |  |  |  |
| TCGA.NH.A50V.01A.11R.A28H.07 | 0.19495674 |  |  |  |
| TCGA.A6.3810.01A.01R.1022.07 | -0.104556209 |  |  |  |
| TCGA.CM.5861.01A.01R.1653.07 | 0.447429718 |  |  |  |
| TCGA.AA.3492.01A.01R.1410.07 | -0.13615258 |  |  |  |
| TCGA.G4.6304.01A.11R.1928.07 | -0.078603472 |  |  |  |
| TCGA.DM.A1D7.01A.11R.A155.07 | 0.010440164 |  |  |  |
| TCGA.G4.6314.01A.11R.1723.07 | 0.579641269 |  |  |  |
| TCGA.AA.3561.01A.01R.0821.07 | -0.113416095 |  |  |  |
| TCGA.AA.A00U.01A.01R.A002.07 | 0.107241212 |  |  |  |
| TCGA.AA.A02K.01A.03R.A32Y.07 | 0.020351431 |  |  |  |
| TCGA.DM.A288.01A.11R.A16W.07 | 0.166461141 |  |  |  |
| TCGA.A6.3809.01A.01R.A278.07 | -0.696022231 |  |  |  |
| TCGA.F4.6809.01A.11R.1839.07 | 0.10318269 |  |  |  |
| TCGA.CM.6675.01A.11R.1839.07 | 0.491012027 |  |  |  |
| TCGA.DM.A0X9.01A.11R.A155.07 | -0.093204535 |  |  |  |
| TCGA.CM.5341.01A.01R.1410.07 | -0.875881067 |  |  |  |
| TCGA.A6.3807.01A.01R.1022.07 | 0.327534938 |  |  |  |
| TCGA.AA.3489.01A.21R.1839.07 | -1.488401856 |  |  |  |
| TCGA.AY.A69D.01A.11R.A37K.07 | 0.259162008 |  |  |  |
| TCGA.A6.2672.01B.03R.2302.07 | -0.751170029 |  |  |  |
| TCGA.AM.5820.01A.01R.1653.07 | 0.570337099 |  |  |  |
| TCGA.A6.3810.01B.04R.A277.07 | -0.812305084 |  |  |  |
| TCGA.D5.6539.01A.11R.1723.07 | 0.005026563 |  |  |  |
| TCGA.SS.A7HO.01A.21R.A37K.07 | 0.239676675 |  |  |  |
| TCGA.AA.3520.01A.01R.0821.07 | 0.242477562 |  |  |  |
| TCGA.AA.3947.01A.01R.1022.07 | -0.293245941 |  |  |  |
| TCGA.AA.3864.01A.01R.1022.07 | 0.376055113 |  |  |  |
| TCGA.AA.3552.01A.01R.0821.07 | -0.388469773 |  |  |  |
| TCGA.AA.3845.01A.01R.1022.07 | 0.119466338 |  |  |  |
| TCGA.AA.A00N.01A.02R.A00A.07 | 0.393046978 |  |  |  |
| TCGA.AA.3688.01A.01R.0905.07 | -0.131509225 |  |  |  |
| TCGA.D5.6532.01A.11R.1723.07 | 0.014589732 |  |  |  |
| TCGA.AA.A01R.01A.21R.A083.07 | -0.518149074 |  |  |  |
| TCGA.AA.A01X.01A.21R.A083.07 | 0.159719288 |  |  |  |
| TCGA.AA.3521.01A.01R.0821.07 | 0.506921334 |  |  |  |
| TCGA.5M.AATE.01A.11R.A41B.07 | 0.183562366 |  |  |  |
| TCGA.DM.A28K.01A.21R.A32Y.07 | 0.089711398 |  |  |  |
| TCGA.AA.3496.01A.21R.1839.07 | -0.222428063 |  |  |  |
| TCGA.AA.3980.01A.02R.1022.07 | -0.493739955 |  |  |  |
| TCGA.CM.6165.01A.11R.1653.07 | 0.28009625 |  |  |  |
| TCGA.F4.6459.01A.11R.1774.07 | 0.482252745 |  |  |  |
| TCGA.A6.2685.01A.01R.1410.07 | -0.298691367 |  |  |  |
| TCGA.AA.A02E.01A.01R.A00A.07 | 0.132359927 |  |  |  |
| TCGA.DM.A28G.01A.11R.A16W.07 | 0.069043703 |  |  |  |
| TCGA.AY.6386.01A.21R.1723.07 | 0.155725177 |  |  |  |
| TCGA.A6.6138.01A.11R.1774.07 | -0.139093945 |  |  |  |
| TCGA.F4.6463.01A.11R.1723.07 | 0.667429909 |  |  |  |
| TCGA.AA.3819.01A.01R.0905.07 | -0.008534958 |  |  |  |
| TCGA.F4.6806.01A.11R.1839.07 | 0.524695416 |  |  |  |
| TCGA.AA.3966.01A.01R.1113.07 | -0.536577615 |  |  |  |
| TCGA.AA.A03J.01A.21R.A16W.07 | -0.109481142 |  |  |  |
| TCGA.AA.3524.01A.02R.0821.07 | 0.304396793 |  |  |  |
| TCGA.AA.3519.01A.02R.0821.07 | -0.048948093 |  |  |  |
| TCGA.CM.6161.01A.11R.1653.07 | -0.021760853 |  |  |  |
| TCGA.AA.3867.01A.01R.1022.07 | 0.507106624 |  |  |  |
| TCGA.AA.3684.01A.02R.0905.07 | -0.148925184 |  |  |  |
| TCGA.AY.4071.01A.01R.1113.07 | -0.028078317 |  |  |  |
| TCGA.D5.7000.01A.11R.A32Z.07 | 0.433263357 |  |  |  |
| TCGA.A6.5660.01A.01R.1653.07 | 0.472662705 |  |  |  |
| TCGA.A6.5662.01A.01R.1653.07 | 0.50031719 |  |  |  |
| TCGA.QG.A5Z2.01A.11R.A28H.07 | -0.24215005 |  |  |  |
| TCGA.A6.2676.01A.01R.0826.07 | -0.592320887 |  |  |  |
| TCGA.AA.3560.01A.01R.0821.07 | -0.138735654 |  |  |  |
| TCGA.NH.A8F7.01A.11R.A41B.07 | 0.179542193 |  |  |  |
| TCGA.AA.A01Q.01A.01R.A002.07 | -0.344292495 |  |  |  |
| TCGA.CM.6172.01A.11R.1653.07 | 0.090806793 |  |  |  |
| TCGA.AA.3956.01A.02R.1022.07 | 0.337667712 |  |  |  |
| TCGA.AA.3655.01A.02R.1723.07 | 0.346694812 |  |  |  |
| TCGA.D5.6922.01A.11R.1928.07 | 0.238786816 |  |  |  |
| TCGA.AY.6196.01A.11R.1723.07 | -4.054282791 |  |  |  |
| TCGA.NH.A6GB.01A.11R.A37K.07 | -0.083820728 |  |  |  |
| TCGA.CA.6715.01A.21R.1839.07 | 0.389402509 |  |  |  |
| TCGA.F4.6856.01A.11R.1928.07 | 0.283053609 |  |  |  |
| TCGA.A6.2679.01A.02R.1410.07 | -0.061467064 |  |  |  |
| TCGA.G4.6586.01A.11R.1774.07 | -0.231577127 |  |  |  |
| TCGA.AA.3856.01A.01R.0905.07 | -0.516841206 |  |  |  |
| TCGA.AZ.4315.01A.01R.1410.07 | -0.04379136 |  |  |  |
| TCGA.CK.4952.01A.01R.1723.07 | 0.017712183 |  |  |  |
| TCGA.5M.AATA.01A.31R.A41B.07 | 0.129388715 |  |  |  |
| TCGA.AA.3848.01A.01R.0905.07 | -0.018768573 |  |  |  |
| TCGA.DM.A28F.01A.11R.A32Y.07 | 0.0582364 |  |  |  |
| TCGA.AY.A54L.01A.11R.A28H.07 | 0.315803221 |  |  |  |
| TCGA.CM.6677.01A.11R.1839.07 | 0.204813859 |  |  |  |
| TCGA.A6.3809.01B.04R.A277.07 | -1.101690914 |  |  |  |
| TCGA.AA.3553.01A.01R.0821.07 | 0.160873156 |  |  |  |
| TCGA.AU.6004.01A.11R.1723.07 | -0.021141238 |  |  |  |
| TCGA.CK.4948.01B.11R.1653.07 | 0.493062447 |  |  |  |
| TCGA.AZ.6606.01A.11R.1839.07 | 0.067883984 |  |  |  |
| TCGA.A6.A565.01A.31R.A28H.07 | -0.448473533 |  |  |  |
| TCGA.AA.A022.01A.21R.A16W.07 | -0.449675822 |  |  |  |
| TCGA.AA.3710.01A.01R.1022.07 | -0.753061909 |  |  |  |
| TCGA.AA.3811.01A.01R.1022.07 | -0.128878128 |  |  |  |
| TCGA.A6.6781.01A.22R.A278.07 | -0.876887742 |  |  |  |
| TCGA.AA.3542.01A.02R.1873.07 | -0.075809754 |  |  |  |
| TCGA.D5.6541.01A.11R.1723.07 | -0.159734473 |  |  |  |
| TCGA.AA.A017.01A.01R.A00A.07 | 0.305804989 |  |  |  |
| TCGA.F4.6808.01A.11R.1839.07 | 0.200893352 |  |  |  |
| TCGA.AA.3851.01A.01R.1022.07 | 0.022176684 |  |  |  |
| TCGA.CM.4747.01A.01R.1410.07 | 0.407859072 |  |  |  |
| TCGA.NH.A6GC.01A.12R.A41B.07 | 0.60245734 |  |  |  |
| TCGA.D5.6930.01A.11R.1928.07 | 0.175650564 |  |  |  |
| TCGA.G4.6307.01A.11R.1723.07 | 0.173708325 |  |  |  |
| TCGA.CM.5864.01A.01R.1653.07 | -0.130477373 |  |  |  |
| TCGA.AA.A00E.01A.01R.A002.07 | -0.127692823 |  |  |  |
| TCGA.A6.6780.01B.04R.A277.07 | -1.646034024 |  |  |  |
| TCGA.AA.3846.01A.01R.1022.07 | -0.257512979 |  |  |  |
| TCGA.AA.3837.01A.01R.0905.07 | 0.687831041 |  |  |  |
| TCGA.AZ.4323.01A.21R.1839.07 | -0.967498739 |  |  |  |
| TCGA.D5.6923.01A.11R.A32Z.07 | 0.579616707 |  |  |  |
| TCGA.CK.4950.01A.01R.1723.07 | 0.15313588 |  |  |  |
| TCGA.A6.6650.01B.02R.A277.07 | -0.138953341 |  |  |  |
| TCGA.DM.A1DA.01A.11R.A155.07 | 0.053543641 |  |  |  |
| TCGA.AA.3715.01A.01R.0905.07 | -0.483250828 |  |  |  |
| TCGA.AA.3664.01A.01R.0905.07 | -0.042898559 |  |  |  |
| TCGA.AA.3697.01A.01R.1723.07 | -0.03602835 |  |  |  |
| TCGA.AA.3517.01A.01R.0821.07 | 0.047736555 |  |  |  |
| TCGA.D5.6533.01A.11R.1723.07 | 0.197670535 |  |  |  |
| TCGA.AA.3531.01A.01R.0821.07 | 0.082094798 |  |  |  |
| TCGA.CM.6166.01A.11R.1653.07 | 0.556923684 |  |  |  |
| TCGA.CM.6170.01A.11R.1653.07 | 0.384321986 |  |  |  |
| TCGA.AA.3979.01A.01R.1022.07 | 0.122988235 |  |  |  |
| TCGA.D5.5541.01A.01R.1653.07 | 0.080001437 |  |  |  |
| TCGA.CK.5913.01A.11R.1653.07 | 0.453628808 |  |  |  |
| TCGA.A6.5659.01A.01R.A278.07 | 0.308533364 |  |  |  |
| TCGA.NH.A8F8.01A.72R.A41B.07 | 0.518730881 |  |  |  |
| TCGA.AA.A02O.01A.21R.A16W.07 | 0.012023835 |  |  |  |
| TCGA.AA.3548.01A.01R.1873.07 | 0.059291152 |  |  |  |
| TCGA.AA.3554.01A.01R.0826.07 | -0.216778941 |  |  |  |
| TCGA.A6.5665.01A.01R.1653.07 | -0.071566316 |  |  |  |
| TCGA.AA.A00J.01A.02R.A002.07 | 0.271818629 |  |  |  |
| TCGA.G4.6302.01A.11R.1723.07 | -0.145263118 |  |  |  |
| TCGA.AA.3869.01A.01R.1022.07 | -0.011849589 |  |  |  |
| TCGA.T9.A92H.01A.11R.A37K.07 | 0.122078084 |  |  |  |
| TCGA.A6.6649.01A.11R.1774.07 | 0.188180384 |  |  |  |
| TCGA.AA.3870.01A.01R.1022.07 | 0.155945828 |  |  |  |
| TCGA.AA.3532.01A.01R.0821.07 | -0.003395269 |  |  |  |
| TCGA.AA.3511.01A.21R.1839.07 | 0.576561475 |  |  |  |
| TCGA.AA.3562.01A.02R.0821.07 | 0.115199881 |  |  |  |
| TCGA.AZ.4616.01A.21R.1839.07 | 0.342074479 |  |  |  |
| TCGA.CM.4744.01A.01R.A32Z.07 | -0.164567505 |  |  |  |
| TCGA.AD.5900.01A.11R.1653.07 | 0.062287459 |  |  |  |
| TCGA.AA.A01G.01A.01R.A002.07 | 0.103260091 |  |  |  |
| TCGA.AZ.5403.01A.01R.1653.07 | 0.5997565 |  |  |  |
| TCGA.AA.3949.01A.01R.1022.07 | -0.894096029 |  |  |  |
| TCGA.AA.A01K.01A.01R.A00A.07 | 0.540999958 |  |  |  |
| TCGA.AU.3779.01A.01R.1723.07 | 0.096044167 |  |  |  |
| TCGA.AY.5543.01A.01R.1653.07 | -0.042772035 |  |  |  |
| TCGA.AZ.4614.01A.01R.1410.07 | 0.157566538 |  |  |  |
| TCGA.AD.6889.01A.11R.1928.07 | 0.236945175 |  |  |  |
| TCGA.AA.3973.01A.01R.1022.07 | 0.267675284 |  |  |  |
| TCGA.NH.A6GA.01A.11R.A37K.07 | -0.009893604 |  |  |  |
| TCGA.D5.5538.01A.01R.1653.07 | -0.043939179 |  |  |  |
| TCGA.AA.A00L.01A.01R.A002.07 | 0.048527542 |  |  |  |
| TCGA.AA.A00W.01A.01R.A002.07 | 0.087426459 |  |  |  |
| TCGA.D5.6927.01A.21R.1928.07 | 0.101297095 |  |  |  |
| TCGA.NH.A50T.01A.11R.A28H.07 | 0.136215167 |  |  |  |
| TCGA.AA.3833.01A.01R.0905.07 | 0.398630665 |  |  |  |
| TCGA.DM.A1D9.01A.11R.A155.07 | 0.19020824 |  |  |  |
| TCGA.AM.5821.01A.01R.1653.07 | 0.242052384 |  |  |  |
| TCGA.AA.A00A.01A.01R.A002.07 | 0.264720584 |  |  |  |
| TCGA.DM.A280.01A.12R.A16W.07 | 0.638178675 |  |  |  |
| TCGA.CA.6718.01A.11R.1839.07 | -0.152321823 |  |  |  |
| TCGA.D5.6531.01A.11R.1723.07 | 0.190194333 |  |  |  |
| TCGA.D5.6536.01A.11R.1723.07 | 0.486044454 |  |  |  |
| TCGA.A6.6781.01B.06R.A277.07 | -3.239763438 |  |  |  |
| TCGA.AA.A01F.01A.01R.A002.07 | 0.20194388 |  |  |  |
| TCGA.G4.6299.01A.11R.1774.07 | 0.113437384 |  |  |  |
| TCGA.AA.3982.01A.02R.1022.07 | -0.655149838 |  |  |  |
| TCGA.G4.6322.01A.11R.1723.07 | 0.17332865 |  |  |  |
| TCGA.A6.6140.01A.11R.1774.07 | 0.044257836 |  |  |  |
| TCGA.AA.3850.01A.01R.1022.07 | 0.023054785 |  |  |  |
| TCGA.AA.3543.01A.01R.0826.07 | -0.369079534 |  |  |  |
| TCGA.G4.6309.01A.21R.1839.07 | 0.116197007 |  |  |  |
| TCGA.AA.3530.01A.01R.1022.07 | -0.137508123 |  |  |  |
| TCGA.AZ.6598.01A.11R.1774.07 | -0.008655522 |  |  |  |
| TCGA.AA.3950.01A.02R.1022.07 | -0.562771346 |  |  |  |
| TCGA.A6.3809.01A.01R.1022.07 | -0.411228061 |  |  |  |
| TCGA.CM.6167.01A.11R.1653.07 | -0.509276539 |  |  |  |
| TCGA.AA.A010.01A.01R.A089.07 | 0.003811964 |  |  |  |
| TCGA.AA.3514.01A.02R.0821.07 | 0.191650962 |  |  |  |
| TCGA.AA.3989.01A.01R.1022.07 | 0.073369364 |  |  |  |
| TCGA.AA.3527.01A.01R.0821.07 | -0.03542022 |  |  |  |
| TCGA.CA.5796.01A.01R.1653.07 | 0.013997473 |  |  |  |
| TCGA.AA.3712.01A.21R.1723.07 | 0.598442239 |  |  |  |
| TCGA.AA.A02W.01A.01R.A00A.07 | 0.173133671 |  |  |  |
| TCGA.A6.2684.01C.08R.A277.07 | -1.019572246 |  |  |  |
| TCGA.AA.3841.01A.01R.0905.07 | 0.305192133 |  |  |  |
| TCGA.CM.6678.01A.11R.1839.07 | 0.509880553 |  |  |  |
| TCGA.AD.6963.01A.11R.1928.07 | -0.277728844 |  |  |  |
| TCGA.A6.5665.01B.03R.2302.07 | -0.255889398 |  |  |  |
| TCGA.AD.6901.01A.11R.1928.07 | 0.052585211 |  |  |  |
| TCGA.5M.AAT4.01A.11R.A41B.07 | 0.200711011 |  |  |  |
| TCGA.DM.A0XD.01A.12R.A155.07 | 0.391867851 |  |  |  |
| TCGA.A6.6141.01A.11R.1774.07 | -0.097164403 |  |  |  |
| TCGA.AA.3681.01A.01R.0905.07 | -0.112278299 |  |  |  |
| TCGA.A6.5666.01A.01R.1653.07 | 0.21856076 |  |  |  |
| TCGA.D5.6924.01A.11R.1928.07 | 0.024265562 |  |  |  |
| TCGA.5M.AAT6.01A.11R.A41B.07 | -0.251603446 |  |  |  |
| TCGA.AA.3509.01A.01R.1410.07 | -0.171315162 |  |  |  |
| TCGA.CK.6748.01A.11R.1839.07 | 0.445734417 |  |  |  |
| TCGA.AA.3525.01A.02R.0826.07 | -0.00795866 |  |  |  |
| TCGA.A6.A56B.01A.31R.A28H.07 | 0.709970442 |  |  |  |
| TCGA.AA.3972.01A.01R.1022.07 | 0.219928111 |  |  |  |
| TCGA.A6.2686.01A.01R.A32Z.07 | -0.863912308 |  |  |  |
| TCGA.G4.6311.01A.11R.1723.07 | 0.55658276 |  |  |  |
| TCGA.AA.3861.01A.01R.1022.07 | -0.114706514 |  |  |  |
| TCGA.G4.6625.01A.21R.1774.07 | -0.367206549 |  |  |  |
| TCGA.AD.6965.01A.11R.1928.07 | 0.229699865 |  |  |  |
| TCGA.AA.A00K.01A.02R.A002.07 | 0.151178407 |  |  |  |
| TCGA.A6.6652.01A.11R.1774.07 | 0.190967584 |  |  |  |
| TCGA.A6.A5ZU.01A.11R.A28H.07 | 0.223050892 |  |  |  |
| TCGA.AA.A00O.01A.02R.A089.07 | 0.302302844 |  |  |  |
| TCGA.A6.6650.01A.11R.A278.07 | 0.158839147 |  |  |  |
| TCGA.AA.3549.01A.02R.0821.07 | 0.307221774 |  |  |  |
| TCGA.AA.3970.01A.01R.1022.07 | -0.231834244 |  |  |  |
| TCGA.AA.3930.01A.01R.1022.07 | -0.285039298 |  |  |  |
| TCGA.G4.6627.01A.11R.1774.07 | 0.147803935 |  |  |  |
| TCGA.F4.6704.01A.11R.1839.07 | -0.175888271 |  |  |  |
| TCGA.CM.5349.01A.21R.1723.07 | 0.380159107 |  |  |  |
| TCGA.AA.3518.01A.02R.0826.07 | -0.356638774 |  |  |  |
| TCGA.AA.3815.01A.01R.1022.07 | -0.551793098 |  |  |  |
| TCGA.AA.3526.01A.02R.A32Z.07 | -0.018703335 |  |  |  |
| TCGA.QL.A97D.01A.12R.A41B.07 | -0.218347305 |  |  |  |
| TCGA.AA.3939.01A.01R.1022.07 | 0.354722397 |  |  |  |
| TCGA.AA.3814.01A.01R.0905.07 | 0.118422116 |  |  |  |
| TCGA.AA.A01V.01A.23R.A083.07 | 0.008842925 |  |  |  |
| TCGA.AZ.4313.01A.01R.1410.07 | 0.366390849 |  |  |  |
| TCGA.AA.3872.01A.01R.1022.07 | 0.035241921 |  |  |  |
| TCGA.A6.3810.01A.01R.A278.07 | -0.072650227 |  |  |  |
| TCGA.AD.6964.01A.11R.1928.07 | -1.155801291 |  |  |  |
| TCGA.A6.2681.01A.01R.1410.07 | 0.352206672 |  |  |  |
| TCGA.A6.2671.01A.01R.1410.07 | 0.352029796 |  |  |  |
| TCGA.CA.6719.01A.11R.1839.07 | 0.383968258 |  |  |  |
| TCGA.CM.4751.01A.02R.1839.07 | 0.245950237 |  |  |  |
| TCGA.AA.3692.01A.01R.0905.07 | -0.407345319 |  |  |  |
| TCGA.G4.6294.01A.11R.1774.07 | -0.066823964 |  |  |  |
| TCGA.D5.6540.01A.11R.1723.07 | 0.598664071 |  |  |  |
| TCGA.D5.6898.01A.11R.1928.07 | 0.330806437 |  |  |  |
| TCGA.G4.6297.01A.11R.1723.07 | 0.355215871 |  |  |  |
| TCGA.G4.6317.02A.11R.2066.07 | 0.350652549 |  |  |  |
| TCGA.AA.3821.01A.01R.1022.07 | 0.04567998 |  |  |  |
| TCGA.A6.2674.01A.02R.0821.07 | -1.409843923 |  |  |  |
| TCGA.F4.6854.01A.11R.1928.07 | 0.546298465 |  |  |  |
| TCGA.G4.6295.01A.11R.1723.07 | -0.389177595 |  |  |  |
| TCGA.A6.2684.01A.01R.1410.07 | -0.046086321 |  |  |  |
| TCGA.A6.6650.01A.11R.1774.07 | 0.132022206 |  |  |  |
| TCGA.AA.3696.01A.01R.0905.07 | 0.704771155 |  |  |  |
| TCGA.A6.6780.01A.11R.1839.07 | -0.919868025 |  |  |  |
| TCGA.AA.3866.01A.01R.1022.07 | -0.347550425 |  |  |  |
| TCGA.AA.3862.01A.01R.1022.07 | -0.322627447 |  |  |  |
| TCGA.AA.3855.01A.01R.1022.07 | -0.215500806 |  |  |  |
| TCGA.AA.A01Z.01A.11R.A083.07 | 0.341786849 |  |  |  |
| TCGA.AA.3678.01A.01R.0905.07 | -0.181757802 |  |  |  |
| TCGA.CK.5914.01A.11R.1653.07 | 0.095554108 |  |  |  |
| TCGA.NH.A8F7.06A.31R.A41B.07 | 0.264184486 |  |  |  |
| TCGA.CM.4748.01A.01R.1410.07 | 0.184741768 |  |  |  |
| TCGA.AY.6197.01A.11R.1723.07 | 0.103822245 |  |  |  |
| TCGA.A6.6781.01A.22R.1928.07 | -1.075479274 |  |  |  |
| TCGA.DM.A1D4.01A.21R.A155.07 | 0.04769087 |  |  |  |
| TCGA.AA.3968.01A.01R.1022.07 | 0.286783047 |  |  |  |
| TCGA.AA.3713.01A.21R.1723.07 | -0.32358906 |  |  |  |
| TCGA.CA.5797.01A.01R.1653.07 | 0.480383904 |  |  |  |
| TCGA.G4.6310.01A.11R.1723.07 | 0.65888246 |  |  |  |
| TCGA.A6.5657.01A.01R.A32Z.07 | 0.506212112 |  |  |  |
| TCGA.A6.5667.01A.21R.1723.07 | 0.771850375 |  |  |  |
| TCGA.NH.A50U.01A.33R.A37K.07 | 0.056480188 |  |  |  |
| TCGA.AA.A02J.01A.01R.A00A.07 | 0.364413323 |  |  |  |
| TCGA.A6.2682.01A.01R.1410.07 | 0.458176402 |  |  |  |
| TCGA.D5.6932.01A.11R.1928.07 | 0.468070932 |  |  |  |
| TCGA.D5.6929.01A.31R.1928.07 | 0.273282953 |  |  |  |
| TCGA.A6.2675.01A.02R.1723.07 | 0.056451109 |  |  |  |
| TCGA.A6.2684.01A.01R.A278.07 | -0.07622253 |  |  |  |
| TCGA.A6.6654.01A.21R.1839.07 | -1.105109025 |  |  |  |
| TCGA.A6.A567.01A.31R.A28H.07 | 0.4542169 |  |  |  |
| TCGA.AA.3666.01A.02R.0905.07 | -0.16199298 |  |  |  |
| TCGA.DM.A1HA.01A.11R.A155.07 | 0.315545233 |  |  |  |
| TCGA.CA.6717.01A.11R.1839.07 | -0.42973951 |  |  |  |
| TCGA.CM.6163.01A.11R.1653.07 | -0.194472936 |  |  |  |
| TCGA.CM.5862.01A.01R.1653.07 | 0.335011533 |  |  |  |
| TCGA.D5.6534.01A.21R.1928.07 | -2.165632535 |  |  |  |
| TCGA.A6.6137.01A.11R.1774.07 | -0.172033207 |  |  |  |
| TCGA.AZ.6605.01A.11R.1839.07 | -0.264211629 |  |  |  |
| TCGA.AA.3984.01A.02R.1022.07 | 0.472584719 |  |  |  |
| TCGA.CA.6716.01A.11R.1839.07 | 0.817516564 |  |  |  |
| TCGA.AA.A01I.01A.02R.A089.07 | 0.023377017 |  |  |  |
| TCGA.AA.3495.01A.01R.1410.07 | -0.249742734 |  |  |  |
| TCGA.AA.A02F.01A.01R.A089.07 | 0.753391415 |  |  |  |
| TCGA.G4.6626.01A.11R.1774.07 | 0.159348296 |  |  |  |
| TCGA.AA.A02Y.01A.43R.A32Y.07 | 0.065030575 |  |  |  |
| TCGA.CM.6674.01A.11R.1839.07 | 0.373441415 |  |  |  |
| TCGA.A6.5656.01B.02R.A277.07 | -1.158154076 |  |  |  |
| TCGA.AA.3693.01A.01R.0905.07 | 0.018769937 |  |  |  |
| TCGA.D5.6931.01A.11R.1928.07 | 0.284957231 |  |  |  |
| TCGA.AA.3556.01A.01R.0821.07 | -0.038151854 |  |  |  |
| TCGA.AZ.4615.01A.01R.1410.07 | -0.099666021 |  |  |  |
| TCGA.AA.3941.01A.01R.1022.07 | 0.084921844 |  |  |  |
| TCGA.D5.6538.01A.11R.1723.07 | 0.258255632 |  |  |  |
| TCGA.AY.A71X.01A.12R.A37K.07 | 0.304277154 |  |  |  |
| TCGA.AA.3858.01A.01R.0905.07 | 0.424043439 |  |  |  |
| TCGA.AA.A02H.01A.01R.A089.07 | 0.607093046 |  |  |  |
| TCGA.D5.6928.01A.11R.1928.07 | -3.188334305 |  |  |  |
| TCGA.AA.3818.01A.01R.0905.07 | 0.14985141 |  |  |  |
| TCGA.AA.A01C.01A.01R.A00A.07 | 0.683430369 |  |  |  |
| TCGA.CM.6164.01A.11R.1653.07 | 0.268141313 |  |  |  |
| TCGA.AA.A00Z.01A.01R.A002.07 | -0.015109095 |  |  |  |
| TCGA.AA.3534.01A.01R.0821.07 | 0.211344422 |  |  |  |
| TCGA.AA.3672.01A.01R.0905.07 | -0.155151025 |  |  |  |
| TCGA.A6.2678.01A.01R.0821.07 | 0.157680172 |  |  |  |
| TCGA.AA.3510.01A.01R.1410.07 | -0.401297408 |  |  |  |
| TCGA.DM.A1D6.01A.21R.A155.07 | 0.31881486 |  |  |  |
| TCGA.CM.6680.01A.11R.1839.07 | 0.107785892 |  |  |  |
| TCGA.AA.3875.01A.01R.0905.07 | -0.302834948 |  |  |  |
| TCGA.CM.5348.01A.21R.1723.07 | -0.127331924 |  |  |  |
| TCGA.CM.6171.01A.11R.1653.07 | 0.135649737 |  |  |  |
| TCGA.A6.2677.01A.01R.0821.07 | 0.111532407 |  |  |  |
| TCGA.AZ.5407.01A.01R.1723.07 | -0.13627208 |  |  |  |
| TCGA.AA.3977.01A.01R.1022.07 | 0.205604402 |  |  |  |
| TCGA.DM.A285.01A.11R.A16W.07 | 0.539931787 |  |  |  |
| TCGA.AZ.6599.01A.11R.1774.07 | 0.213234663 |  |  |  |
| TCGA.QG.A5YX.01A.11R.A28H.07 | 0.048953648 |  |  |  |
| TCGA.A6.3808.01A.01R.1022.07 | -0.107980891 |  |  |  |
| TCGA.DM.A1DB.01A.11R.A155.07 | 0.010935123 |  |  |  |
| TCGA.AA.3529.01A.02R.0821.07 | 0.126793232 |  |  |  |
| TCGA.WS.AB45.01A.11R.A41B.07 | -3.286445413 |  |  |  |
| TCGA.AA.A01P.01A.21R.A083.07 | -0.454264859 |  |  |  |
| TCGA.AA.3502.01A.01R.1410.07 | -0.158002738 |  |  |  |
| TCGA.CA.5254.01A.21R.1839.07 | 0.423848592 |  |  |  |
| TCGA.D5.5537.01A.21R.1928.07 | 0.612081156 |  |  |  |
| TCGA.A6.5661.01B.05R.2302.07 | -0.636754956 |  |  |  |
| TCGA.QG.A5YW.01A.11R.A28H.07 | -0.15778774 |  |  |  |
| TCGA.NH.A5IV.01A.42R.A37K.07 | -0.524682993 |  |  |  |
| TCGA.A6.5661.01A.01R.1653.07 | 0.26911508 |  |  |  |
| TCGA.D5.6920.01A.11R.1928.07 | 0.044485872 |  |  |  |
| TCGA.CK.4947.01B.11R.1653.07 | 0.004166341 |  |  |  |
| TCGA.QG.A5YV.01A.11R.A28H.07 | 0.041639713 |  |  |  |
| TCGA.A6.2683.01A.01R.0821.07 | 0.091664041 |  |  |  |
| TCGA.AA.A024.01A.02R.A00A.07 | 0.345005655 |  |  |  |
| TCGA.A6.2680.01A.01R.1410.07 | 0.243056936 |  |  |  |
| TCGA.AZ.6603.01A.11R.1839.07 | 0.230704194 |  |  |  |
| TCGA.F4.6807.01A.11R.1839.07 | -0.349095034 |  |  |  |
| TCGA.AA.3842.01A.01R.1022.07 | 0.430013828 |  |  |  |
| TCGA.A6.6651.01A.21R.1839.07 | -1.005450105 |  |  |  |
| TCGA.AA.3667.01A.01R.0905.07 | -0.017199544 |  |  |  |
| TCGA.D5.6926.01A.11R.1928.07 | 0.280873716 |  |  |  |
| TCGA.AY.A8YK.01A.11R.A41B.07 | 0.056951181 |  |  |  |
| TCGA.CM.6676.01A.11R.1839.07 | 0.658934235 |  |  |  |
| TCGA.AD.6899.01A.11R.1928.07 | -0.068245887 |  |  |  |
| TCGA.AA.3986.01A.02R.1022.07 | -0.640078221 |  |  |  |
| TCGA.AA.A01T.01A.21R.A16W.07 | 0.151705913 |  |  |  |
| TCGA.A6.5656.01A.21R.1839.07 | 0.118352062 |  |  |  |
| TCGA.CM.6679.01A.11R.1839.07 | 0.334963391 |  |  |  |
| TCGA.F4.6570.01A.11R.1774.07 | -0.382208429 |  |  |  |
| TCGA.CM.5868.01A.01R.1653.07 | 0.554533611 |  |  |  |
| TCGA.CK.4951.01A.01R.1410.07 | -0.109801292 |  |  |  |
| TCGA.G4.6320.01A.11R.1723.07 | 0.094465866 |  |  |  |
| TCGA.CA.5256.01A.01R.1410.07 | -0.172436172 |  |  |  |
| TCGA.DM.A28E.01A.11R.A32Y.07 | 0.144472726 |  |  |  |
| TCGA.AA.A00Q.01A.01R.A002.07 | -0.019397752 |  |  |  |
| TCGA.D5.5540.01A.01R.1653.07 | 0.034716525 |  |  |  |
| TCGA.G4.6321.01A.11R.1723.07 | 0.02553129 |  |  |  |
| TCGA.G4.6588.01A.11R.1774.07 | 0.301204977 |  |  |  |
| TCGA.AA.3852.01A.01R.0905.07 | 0.274662833 |  |  |  |
| TCGA.AA.3812.01A.01R.0905.07 | 0.254278847 |  |  |  |
| TCGA.3L.AA1B.01A.11R.A37K.07 | 0.079359415 |  |  |  |
| TCGA.AA.3831.01A.01R.0905.07 | -0.145821776 |  |  |  |
| TCGA.DM.A282.01A.12R.A16W.07 | 0.342246552 |  |  |  |
| TCGA.CK.5915.01A.11R.1653.07 | 0.376042174 |  |  |  |
| TCGA.AA.3680.01A.01R.0905.07 | -0.161587105 |  |  |  |
| TCGA.AZ.4308.01A.01R.1410.07 | 0.309042008 |  |  |  |
| TCGA.DM.A28H.01A.11R.A16W.07 | 0.041111987 |  |  |  |
| TCGA.AA.3955.01A.02R.1022.07 | 0.011489745 |  |  |  |
| TCGA.AA.3506.01A.01R.1410.07 | 0.302573358 |  |  |  |
| TCGA.AZ.6600.01A.11R.1774.07 | 0.233308632 |  |  |  |
| TCGA.CM.4752.01A.01R.1410.07 | -0.132888455 |  |  |  |
| TCGA.G4.6315.01A.11R.1723.07 | 0.250708942 |  |  |  |
| TCGA.DM.A1D8.01A.11R.A155.07 | 0.097265943 |  |  |  |
| TCGA.CM.4743.01A.01R.1723.07 | 0.066247065 |  |  |  |
| TCGA.AD.6895.01A.11R.1928.07 | 0.441786404 |  |  |  |
| TCGA.AA.3679.01A.02R.0905.07 | 0.309637563 |  |  |  |
| TCGA.CK.6746.01A.11R.1839.07 | -0.309839378 |  |  |  |
| TCGA.G4.6628.01A.11R.1839.07 | -0.765286751 |  |  |  |
| TCGA.AA.A004.01A.01R.A00A.07 | 0.00657397 |  |  |  |
| TCGA.DM.A28A.01A.21R.A32Y.07 | 0.599426406 |  |  |  |
| TCGA.G4.6317.01A.11R.1723.07 | 0.286801456 |  |  |  |
| TCGA.A6.5664.01A.21R.1839.07 | 0.037858379 |  |  |  |
| TCGA.AA.3952.01A.01R.1022.07 | 0.317706363 |  |  |  |
| TCGA.AZ.6608.01A.11R.1839.07 | 0.19181856 |  |  |  |
| TCGA.CK.6751.01A.11R.1839.07 | 0.654192141 |  |  |  |
| TCGA.AD.A5EK.01A.11R.A28H.07 | 0.117881026 |  |  |  |
