# Supplementary material for: Infiltrating T-cell abundance combined with EMT-related gene expression as a prognostic factor of colon cancer
Source: Bioengineered. 2021 Jun 27;12(1):2688–701. doi: 10.1080/21655979.2021.1939618 (PMC8806648; doi:10.1080/21655979.2021.1939618)
Supplement: Supplemental Material [file KBIE_A_1939618_SM4068.zip › supplementary/Table_S2.docx]

| **Table S2:ITA value adjusted by purity analysis** | | | | | |
| --- | --- | --- | --- | --- | --- |
|  | x |  |  |  |  |
| TCGA.AA.3854.01A.01R.0905.07 | 0.114985482 |  |  |  |  |
| TCGA.CM.6169.01A.11R.1653.07 | -0.76790633 |  |  |  |  |
| TCGA.D5.6530.01A.11R.1723.07 | 0.373593087 |  |  |  |  |
| TCGA.AA.A00F.01A.01R.A002.07 | 0.384054537 |  |  |  |  |
| TCGA.A6.5659.01A.01R.1653.07 | 0.087693568 |  |  |  |  |
| TCGA.AA.3494.01A.01R.1410.07 | 0.265966119 |  |  |  |  |
| TCGA.AA.A029.01A.01R.A00A.07 | 0.042507607 |  |  |  |  |
| TCGA.AD.6888.01A.11R.1928.07 | 0.031864695 |  |  |  |  |
| TCGA.DM.A28M.01A.12R.A16W.07 | -0.060333418 |  |  |  |  |
| TCGA.DM.A1D0.01A.11R.A155.07 | -0.293617296 |  |  |  |  |
| TCGA.AA.3544.01A.01R.1873.07 | -0.48440554 |  |  |  |  |
| TCGA.F4.6805.01A.11R.1839.07 | -0.35810043 |  |  |  |  |
| TCGA.F4.6569.01A.11R.1774.07 | -0.937047183 |  |  |  |  |
| TCGA.CM.5863.01A.21R.1839.07 | -0.239046778 |  |  |  |  |
| TCGA.AA.A01S.01A.21R.A083.07 | -0.233343757 |  |  |  |  |
| TCGA.CM.6168.01A.11R.1653.07 | -0.4890314 |  |  |  |  |
| TCGA.AD.6548.01A.11R.1839.07 | 0.072932259 |  |  |  |  |
| TCGA.AA.A00D.01A.01R.A002.07 | 0.147328306 |  |  |  |  |
| TCGA.CA.5255.01A.11R.1839.07 | 0.271531713 |  |  |  |  |
| TCGA.A6.4105.01A.02R.1774.07 | 0.05048465 |  |  |  |  |
| TCGA.D5.6529.01A.11R.1774.07 | -0.282780522 |  |  |  |  |
| TCGA.AZ.6601.01A.11R.1774.07 | 0.017127528 |  |  |  |  |
| TCGA.G4.6306.01A.11R.1774.07 | 0.524588297 |  |  |  |  |
| TCGA.5M.AAT5.01A.21R.A41B.07 | 0.346021179 |  |  |  |  |
| TCGA.A6.A566.01A.11R.A28H.07 | -1.896421965 |  |  |  |  |
| TCGA.AY.4070.01A.01R.1113.07 | 0.785905115 |  |  |  |  |
| TCGA.AA.3522.01A.01R.0821.07 | 0.454155958 |  |  |  |  |
| TCGA.AA.3663.01A.01R.1723.07 | -0.079813421 |  |  |  |  |
| TCGA.AA.3971.01A.01R.1022.07 | 0.337304623 |  |  |  |  |
| TCGA.A6.2672.01A.01R.0826.07 | -0.071559116 |  |  |  |  |
| TCGA.A6.6780.01A.11R.A278.07 | -0.191839515 |  |  |  |  |
| TCGA.CM.6162.01A.11R.1653.07 | -1.454367884 |  |  |  |  |
| TCGA.CK.6747.01A.11R.1839.07 | 0.196583353 |  |  |  |  |
| TCGA.A6.4107.01A.02R.1410.07 | -0.039459609 |  |  |  |  |
| TCGA.AA.3675.01A.02R.0905.07 | -0.003890177 |  |  |  |  |
| TCGA.F4.6703.01A.11R.1839.07 | -3.080397363 |  |  |  |  |
| TCGA.AA.3877.01A.01R.1022.07 | -0.214704378 |  |  |  |  |
| TCGA.A6.6782.01A.11R.1839.07 | -0.246563972 |  |  |  |  |
| TCGA.A6.6653.01A.11R.1774.07 | -0.205490959 |  |  |  |  |
| TCGA.AA.3860.01A.02R.0905.07 | 0.001568848 |  |  |  |  |
| TCGA.G4.6293.01A.11R.1723.07 | 0.505797881 |  |  |  |  |
| TCGA.G4.6298.01A.11R.1723.07 | -0.227611619 |  |  |  |  |
| TCGA.CM.4746.01A.01R.1410.07 | -0.048735944 |  |  |  |  |
| TCGA.AD.A5EJ.01A.11R.A28H.07 | 0.155441658 |  |  |  |  |
| TCGA.DM.A0XF.01A.11R.A155.07 | 0.318348169 |  |  |  |  |
| TCGA.A6.6648.01A.11R.1774.07 | 0.507009761 |  |  |  |  |
| TCGA.A6.5656.01A.21R.A278.07 | 0.429032351 |  |  |  |  |
| TCGA.AA.3685.01A.02R.A32Z.07 | -0.234648843 |  |  |  |  |
| TCGA.4N.A93T.01A.11R.A37K.07 | 0.547554708 |  |  |  |  |
| TCGA.DM.A28C.01A.11R.A32Y.07 | 0.046738547 |  |  |  |  |
| TCGA.CK.5912.01A.11R.1653.07 | -0.011360698 |  |  |  |  |
| TCGA.AA.3516.01A.02R.0826.07 | 0.013007242 |  |  |  |  |
| TCGA.AD.6890.01A.11R.1928.07 | 0.385540214 |  |  |  |  |
| TCGA.A6.2674.01B.04R.A277.07 | -1.333821926 |  |  |  |  |
| TCGA.F4.6461.01A.11R.1774.07 | -0.061389378 |  |  |  |  |
| TCGA.AA.3662.01A.01R.1723.07 | -0.025475618 |  |  |  |  |
| TCGA.A6.2677.01B.02R.A277.07 | 0.325462404 |  |  |  |  |
| TCGA.AA.3673.01A.01R.0905.07 | 0.550550147 |  |  |  |  |
| TCGA.AA.A02R.01A.01R.A00A.07 | 0.070722801 |  |  |  |  |
| TCGA.D5.6535.01A.11R.1723.07 | 0.342653109 |  |  |  |  |
| TCGA.D5.6537.01A.11R.1723.07 | 0.16894103 |  |  |  |  |
| TCGA.4T.AA8H.01A.11R.A41B.07 | 0.489585938 |  |  |  |  |
| TCGA.AA.3994.01A.01R.1113.07 | 0.04536537 |  |  |  |  |
| TCGA.CK.5916.01A.11R.1653.07 | -0.128708361 |  |  |  |  |
| TCGA.RU.A8FL.01A.11R.A37K.07 | 0.076181783 |  |  |  |  |
| TCGA.F4.6460.01A.11R.1774.07 | 0.243945929 |  |  |  |  |
| TCGA.AA.A00R.01A.01R.A002.07 | 0.193595498 |  |  |  |  |
| TCGA.AA.3555.01A.01R.0821.07 | -0.074892614 |  |  |  |  |
| TCGA.CM.5344.01A.21R.1723.07 | -0.357849547 |  |  |  |  |
| TCGA.F4.6855.01A.11R.1928.07 | -0.526527346 |  |  |  |  |
| TCGA.G4.6323.01A.11R.1723.07 | 0.826014373 |  |  |  |  |
| TCGA.A6.6142.01A.11R.1774.07 | -0.637192566 |  |  |  |  |
| TCGA.AA.3844.01A.01R.1022.07 | 0.292520992 |  |  |  |  |
| TCGA.G4.6303.01A.11R.1774.07 | 0.203535929 |  |  |  |  |
| TCGA.DM.A1HB.01A.21R.A180.07 | -0.377763531 |  |  |  |  |
| TCGA.A6.5659.01B.04R.A277.07 | -0.465998748 |  |  |  |  |
| TCGA.D5.5539.01A.01R.1653.07 | -0.2663347 |  |  |  |  |
| TCGA.AA.3538.01A.01R.0821.07 | 0.052887327 |  |  |  |  |
| TCGA.AA.A03F.01A.11R.A16W.07 | 0.254192651 |  |  |  |  |
| TCGA.AA.3660.01A.01R.1723.07 | 0.192887832 |  |  |  |  |
| TCGA.CM.5860.01A.01R.1653.07 | -0.603354308 |  |  |  |  |
| TCGA.AA.3975.01A.01R.1022.07 | 0.343123105 |  |  |  |  |
| TCGA.A6.2674.01A.02R.A278.07 | -1.182795183 |  |  |  |  |
| TCGA.AZ.6607.01A.11R.1839.07 | -0.574943623 |  |  |  |  |
| TCGA.NH.A50V.01A.11R.A28H.07 | 0.297135192 |  |  |  |  |
| TCGA.A6.3810.01A.01R.1022.07 | -0.524963887 |  |  |  |  |
| TCGA.CM.5861.01A.01R.1653.07 | 0.013442882 |  |  |  |  |
| TCGA.AA.3492.01A.01R.1410.07 | -0.157477733 |  |  |  |  |
| TCGA.G4.6304.01A.11R.1928.07 | 0.380222502 |  |  |  |  |
| TCGA.DM.A1D7.01A.11R.A155.07 | 0.283845345 |  |  |  |  |
| TCGA.G4.6314.01A.11R.1723.07 | -0.053071846 |  |  |  |  |
| TCGA.AA.3561.01A.01R.0821.07 | -0.269910884 |  |  |  |  |
| TCGA.AA.A00U.01A.01R.A002.07 | 0.55613321 |  |  |  |  |
| TCGA.AA.A02K.01A.03R.A32Y.07 | 0.070710514 |  |  |  |  |
| TCGA.DM.A288.01A.11R.A16W.07 | -0.6597557 |  |  |  |  |
| TCGA.A6.3809.01A.01R.A278.07 | -0.437910352 |  |  |  |  |
| TCGA.F4.6809.01A.11R.1839.07 | 0.232328946 |  |  |  |  |
| TCGA.CM.6675.01A.11R.1839.07 | 0.497141841 |  |  |  |  |
| TCGA.DM.A0X9.01A.11R.A155.07 | 0.403622672 |  |  |  |  |
| TCGA.CM.5341.01A.01R.1410.07 | -1.448340457 |  |  |  |  |
| TCGA.A6.3807.01A.01R.1022.07 | -0.005775194 |  |  |  |  |
| TCGA.AA.3489.01A.21R.1839.07 | -1.355593899 |  |  |  |  |
| TCGA.AY.A69D.01A.11R.A37K.07 | 0.445677508 |  |  |  |  |
| TCGA.A6.2672.01B.03R.2302.07 | 0.519477713 |  |  |  |  |
| TCGA.AM.5820.01A.01R.1653.07 | -0.433113407 |  |  |  |  |
| TCGA.A6.3810.01B.04R.A277.07 | -0.316772583 |  |  |  |  |
| TCGA.D5.6539.01A.11R.1723.07 | 0.282693716 |  |  |  |  |
| TCGA.SS.A7HO.01A.21R.A37K.07 | 0.294665898 |  |  |  |  |
| TCGA.AA.3520.01A.01R.0821.07 | -0.067498471 |  |  |  |  |
| TCGA.AA.3947.01A.01R.1022.07 | -0.830327656 |  |  |  |  |
| TCGA.AA.3864.01A.01R.1022.07 | 0.035182283 |  |  |  |  |
| TCGA.AA.3552.01A.01R.0821.07 | 0.386366029 |  |  |  |  |
| TCGA.AA.3845.01A.01R.1022.07 | 0.303114005 |  |  |  |  |
| TCGA.AA.A00N.01A.02R.A00A.07 | -0.251034551 |  |  |  |  |
| TCGA.AA.3688.01A.01R.0905.07 | 0.257670319 |  |  |  |  |
| TCGA.D5.6532.01A.11R.1723.07 | 0.08706719 |  |  |  |  |
| TCGA.AA.A01R.01A.21R.A083.07 | 0.278770901 |  |  |  |  |
| TCGA.AA.A01X.01A.21R.A083.07 | 0.351204817 |  |  |  |  |
| TCGA.AA.3521.01A.01R.0821.07 | 0.040861036 |  |  |  |  |
| TCGA.5M.AATE.01A.11R.A41B.07 | 0.389619469 |  |  |  |  |
| TCGA.DM.A28K.01A.21R.A32Y.07 | -0.062677506 |  |  |  |  |
| TCGA.AA.3496.01A.21R.1839.07 | -0.231528845 |  |  |  |  |
| TCGA.AA.3980.01A.02R.1022.07 | 0.223813306 |  |  |  |  |
| TCGA.CM.6165.01A.11R.1653.07 | -0.040064125 |  |  |  |  |
| TCGA.F4.6459.01A.11R.1774.07 | -0.104096052 |  |  |  |  |
| TCGA.A6.2685.01A.01R.1410.07 | -0.699478017 |  |  |  |  |
| TCGA.AA.A02E.01A.01R.A00A.07 | 0.585102602 |  |  |  |  |
| TCGA.DM.A28G.01A.11R.A16W.07 | 0.505945796 |  |  |  |  |
| TCGA.AY.6386.01A.21R.1723.07 | 0.368795914 |  |  |  |  |
| TCGA.A6.6138.01A.11R.1774.07 | -0.154971497 |  |  |  |  |
| TCGA.F4.6463.01A.11R.1723.07 | 0.224494413 |  |  |  |  |
| TCGA.AA.3819.01A.01R.0905.07 | 0.270089238 |  |  |  |  |
| TCGA.F4.6806.01A.11R.1839.07 | 0.321760531 |  |  |  |  |
| TCGA.AA.3966.01A.01R.1113.07 | -0.52405827 |  |  |  |  |
| TCGA.AA.A03J.01A.21R.A16W.07 | 0.070010869 |  |  |  |  |
| TCGA.AA.3524.01A.02R.0821.07 | 0.019674235 |  |  |  |  |
| TCGA.AA.3519.01A.02R.0821.07 | 0.089959649 |  |  |  |  |
| TCGA.CM.6161.01A.11R.1653.07 | 0.525796534 |  |  |  |  |
| TCGA.AA.3867.01A.01R.1022.07 | -0.141626556 |  |  |  |  |
| TCGA.AA.3684.01A.02R.0905.07 | -0.51934347 |  |  |  |  |
| TCGA.AY.4071.01A.01R.1113.07 | 0.223159036 |  |  |  |  |
| TCGA.D5.7000.01A.11R.A32Z.07 | 0.283811638 |  |  |  |  |
| TCGA.A6.5660.01A.01R.1653.07 | -0.094820744 |  |  |  |  |
| TCGA.A6.5662.01A.01R.1653.07 | -0.280702688 |  |  |  |  |
| TCGA.QG.A5Z2.01A.11R.A28H.07 | 1.148014487 |  |  |  |  |
| TCGA.A6.2676.01A.01R.0826.07 | -0.238860424 |  |  |  |  |
| TCGA.AA.3560.01A.01R.0821.07 | -0.189440922 |  |  |  |  |
| TCGA.NH.A8F7.01A.11R.A41B.07 | 0.003293991 |  |  |  |  |
| TCGA.AA.A01Q.01A.01R.A002.07 | 0.343967512 |  |  |  |  |
| TCGA.CM.6172.01A.11R.1653.07 | 0.039897397 |  |  |  |  |
| TCGA.AA.3956.01A.02R.1022.07 | 0.332870309 |  |  |  |  |
| TCGA.AA.3655.01A.02R.1723.07 | 0.31655666 |  |  |  |  |
| TCGA.D5.6922.01A.11R.1928.07 | 0.125747798 |  |  |  |  |
| TCGA.AY.6196.01A.11R.1723.07 | -3.550372831 |  |  |  |  |
| TCGA.NH.A6GB.01A.11R.A37K.07 | 0.473621889 |  |  |  |  |
| TCGA.CA.6715.01A.21R.1839.07 | -0.053644044 |  |  |  |  |
| TCGA.F4.6856.01A.11R.1928.07 | 0.432753969 |  |  |  |  |
| TCGA.A6.2679.01A.02R.1410.07 | 0.175983655 |  |  |  |  |
| TCGA.G4.6586.01A.11R.1774.07 | 0.526479209 |  |  |  |  |
| TCGA.AA.3856.01A.01R.0905.07 | 0.387981671 |  |  |  |  |
| TCGA.AZ.4315.01A.01R.1410.07 | -0.313126684 |  |  |  |  |
| TCGA.CK.4952.01A.01R.1723.07 | -0.060404581 |  |  |  |  |
| TCGA.5M.AATA.01A.31R.A41B.07 | 0.458257193 |  |  |  |  |
| TCGA.AA.3848.01A.01R.0905.07 | 0.193471103 |  |  |  |  |
| TCGA.DM.A28F.01A.11R.A32Y.07 | 0.36863956 |  |  |  |  |
| TCGA.AY.A54L.01A.11R.A28H.07 | 0.417580039 |  |  |  |  |
| TCGA.CM.6677.01A.11R.1839.07 | 0.086124387 |  |  |  |  |
| TCGA.A6.3809.01B.04R.A277.07 | -0.674237398 |  |  |  |  |
| TCGA.AA.3553.01A.01R.0821.07 | 0.025668259 |  |  |  |  |
| TCGA.AU.6004.01A.11R.1723.07 | 0.02722228 |  |  |  |  |
| TCGA.CK.4948.01B.11R.1653.07 | 0.031412199 |  |  |  |  |
| TCGA.AZ.6606.01A.11R.1839.07 | 0.449979181 |  |  |  |  |
| TCGA.A6.A565.01A.31R.A28H.07 | 0.182116768 |  |  |  |  |
| TCGA.AA.A022.01A.21R.A16W.07 | 0.523718359 |  |  |  |  |
| TCGA.AA.3710.01A.01R.1022.07 | -0.185403546 |  |  |  |  |
| TCGA.AA.3811.01A.01R.1022.07 | 0.477052511 |  |  |  |  |
| TCGA.A6.6781.01A.22R.A278.07 | -1.155540082 |  |  |  |  |
| TCGA.AA.3542.01A.02R.1873.07 | -0.758838109 |  |  |  |  |
| TCGA.D5.6541.01A.11R.1723.07 | -0.722150541 |  |  |  |  |
| TCGA.AA.A017.01A.01R.A00A.07 | 0.207539581 |  |  |  |  |
| TCGA.F4.6808.01A.11R.1839.07 | 0.012067512 |  |  |  |  |
| TCGA.AA.3851.01A.01R.1022.07 | 0.169852015 |  |  |  |  |
| TCGA.CM.4747.01A.01R.1410.07 | -0.17943888 |  |  |  |  |
| TCGA.NH.A6GC.01A.12R.A41B.07 | 0.047902576 |  |  |  |  |
| TCGA.D5.6930.01A.11R.1928.07 | 0.125472208 |  |  |  |  |
| TCGA.G4.6307.01A.11R.1723.07 | 0.134531563 |  |  |  |  |
| TCGA.CM.5864.01A.01R.1653.07 | 0.181044345 |  |  |  |  |
| TCGA.AA.A00E.01A.01R.A002.07 | 0.386493236 |  |  |  |  |
| TCGA.A6.6780.01B.04R.A277.07 | -1.036316424 |  |  |  |  |
| TCGA.AA.3846.01A.01R.1022.07 | 0.345358216 |  |  |  |  |
| TCGA.AA.3837.01A.01R.0905.07 | 0.335039439 |  |  |  |  |
| TCGA.AZ.4323.01A.21R.1839.07 | 0.355370373 |  |  |  |  |
| TCGA.D5.6923.01A.11R.A32Z.07 | -0.078629908 |  |  |  |  |
| TCGA.CK.4950.01A.01R.1723.07 | 0.291523293 |  |  |  |  |
| TCGA.A6.6650.01B.02R.A277.07 | 0.676144953 |  |  |  |  |
| TCGA.DM.A1DA.01A.11R.A155.07 | 0.060554179 |  |  |  |  |
| TCGA.AA.3715.01A.01R.0905.07 | -0.633223558 |  |  |  |  |
| TCGA.AA.3664.01A.01R.0905.07 | 0.632369708 |  |  |  |  |
| TCGA.AA.3697.01A.01R.1723.07 | 0.308162705 |  |  |  |  |
| TCGA.AA.3517.01A.01R.0821.07 | 0.303869484 |  |  |  |  |
| TCGA.D5.6533.01A.11R.1723.07 | 0.053207157 |  |  |  |  |
| TCGA.AA.3531.01A.01R.0821.07 | -0.097918252 |  |  |  |  |
| TCGA.CM.6166.01A.11R.1653.07 | 0.124545293 |  |  |  |  |
| TCGA.CM.6170.01A.11R.1653.07 | 0.106222781 |  |  |  |  |
| TCGA.AA.3979.01A.01R.1022.07 | -0.194544124 |  |  |  |  |
| TCGA.D5.5541.01A.01R.1653.07 | -0.052257365 |  |  |  |  |
| TCGA.CK.5913.01A.11R.1653.07 | 0.000225082 |  |  |  |  |
| TCGA.A6.5659.01A.01R.A278.07 | -0.017175111 |  |  |  |  |
| TCGA.NH.A8F8.01A.72R.A41B.07 | -0.049421788 |  |  |  |  |
| TCGA.AA.A02O.01A.21R.A16W.07 | 0.346240797 |  |  |  |  |
| TCGA.AA.3548.01A.01R.1873.07 | 0.297305121 |  |  |  |  |
| TCGA.AA.3554.01A.01R.0826.07 | -0.396083574 |  |  |  |  |
| TCGA.A6.5665.01A.01R.1653.07 | 0.750053471 |  |  |  |  |
| TCGA.AA.A00J.01A.02R.A002.07 | 0.292898794 |  |  |  |  |
| TCGA.G4.6302.01A.11R.1723.07 | -0.692106036 |  |  |  |  |
| TCGA.AA.3869.01A.01R.1022.07 | -0.03940337 |  |  |  |  |
| TCGA.T9.A92H.01A.11R.A37K.07 | 0.307910752 |  |  |  |  |
| TCGA.A6.6649.01A.11R.1774.07 | 0.390893341 |  |  |  |  |
| TCGA.AA.3870.01A.01R.1022.07 | -0.182135289 |  |  |  |  |
| TCGA.AA.3532.01A.01R.0821.07 | -0.130214367 |  |  |  |  |
| TCGA.AA.3511.01A.21R.1839.07 | -0.066556005 |  |  |  |  |
| TCGA.AA.3562.01A.02R.0821.07 | 0.114385751 |  |  |  |  |
| TCGA.AZ.4616.01A.21R.1839.07 | 0.18361156 |  |  |  |  |
| TCGA.CM.4744.01A.01R.A32Z.07 | 0.163942608 |  |  |  |  |
| TCGA.AD.5900.01A.11R.1653.07 | 0.121369234 |  |  |  |  |
| TCGA.AA.A01G.01A.01R.A002.07 | 0.454501976 |  |  |  |  |
| TCGA.AZ.5403.01A.01R.1653.07 | -0.470261242 |  |  |  |  |
| TCGA.AA.3949.01A.01R.1022.07 | -0.914978192 |  |  |  |  |
| TCGA.AA.A01K.01A.01R.A00A.07 | 0.162704552 |  |  |  |  |
| TCGA.AU.3779.01A.01R.1723.07 | 0.044517969 |  |  |  |  |
| TCGA.AY.5543.01A.01R.1653.07 | 0.285616815 |  |  |  |  |
| TCGA.AZ.4614.01A.01R.1410.07 | 0.360275471 |  |  |  |  |
| TCGA.AD.6889.01A.11R.1928.07 | 0.276252279 |  |  |  |  |
| TCGA.AA.3973.01A.01R.1022.07 | 0.312731152 |  |  |  |  |
| TCGA.NH.A6GA.01A.11R.A37K.07 | 0.695637131 |  |  |  |  |
| TCGA.D5.5538.01A.01R.1653.07 | -0.402725925 |  |  |  |  |
| TCGA.AA.A00L.01A.01R.A002.07 | 0.128609342 |  |  |  |  |
| TCGA.AA.A00W.01A.01R.A002.07 | 0.710085203 |  |  |  |  |
| TCGA.D5.6927.01A.21R.1928.07 | 0.084626901 |  |  |  |  |
| TCGA.NH.A50T.01A.11R.A28H.07 | -0.219116339 |  |  |  |  |
| TCGA.AA.3833.01A.01R.0905.07 | 0.065283465 |  |  |  |  |
| TCGA.DM.A1D9.01A.11R.A155.07 | 0.316857336 |  |  |  |  |
| TCGA.AM.5821.01A.01R.1653.07 | -0.161496396 |  |  |  |  |
| TCGA.AA.A00A.01A.01R.A002.07 | 0.238836071 |  |  |  |  |
| TCGA.DM.A280.01A.12R.A16W.07 | -0.501709123 |  |  |  |  |
| TCGA.CA.6718.01A.11R.1839.07 | 0.220628982 |  |  |  |  |
| TCGA.D5.6531.01A.11R.1723.07 | -0.065778508 |  |  |  |  |
| TCGA.D5.6536.01A.11R.1723.07 | -0.562624524 |  |  |  |  |
| TCGA.A6.6781.01B.06R.A277.07 | -3.153773654 |  |  |  |  |
| TCGA.AA.A01F.01A.01R.A002.07 | -0.029401127 |  |  |  |  |
| TCGA.G4.6299.01A.11R.1774.07 | 0.410197531 |  |  |  |  |
| TCGA.AA.3982.01A.02R.1022.07 | 0.195980379 |  |  |  |  |
| TCGA.G4.6322.01A.11R.1723.07 | 0.496055064 |  |  |  |  |
| TCGA.A6.6140.01A.11R.1774.07 | 0.492154573 |  |  |  |  |
| TCGA.AA.3850.01A.01R.1022.07 | 0.257050091 |  |  |  |  |
| TCGA.AA.3543.01A.01R.0826.07 | 0.331350211 |  |  |  |  |
| TCGA.G4.6309.01A.21R.1839.07 | 0.550627464 |  |  |  |  |
| TCGA.AA.3530.01A.01R.1022.07 | 0.607195598 |  |  |  |  |
| TCGA.AZ.6598.01A.11R.1774.07 | -0.018187112 |  |  |  |  |
| TCGA.AA.3950.01A.02R.1022.07 | -0.896885886 |  |  |  |  |
| TCGA.A6.3809.01A.01R.1022.07 | -0.091549854 |  |  |  |  |
| TCGA.CM.6167.01A.11R.1653.07 | -0.648531377 |  |  |  |  |
| TCGA.AA.A010.01A.01R.A089.07 | 0.600072325 |  |  |  |  |
| TCGA.AA.3514.01A.02R.0821.07 | -0.413655348 |  |  |  |  |
| TCGA.AA.3989.01A.01R.1022.07 | -0.019368936 |  |  |  |  |
| TCGA.AA.3527.01A.01R.0821.07 | -0.609596384 |  |  |  |  |
| TCGA.CA.5796.01A.01R.1653.07 | 0.291733833 |  |  |  |  |
| TCGA.AA.3712.01A.21R.1723.07 | -0.032567584 |  |  |  |  |
| TCGA.AA.A02W.01A.01R.A00A.07 | 0.54341271 |  |  |  |  |
| TCGA.A6.2684.01C.08R.A277.07 | -0.71707192 |  |  |  |  |
| TCGA.AA.3841.01A.01R.0905.07 | 0.086887937 |  |  |  |  |
| TCGA.CM.6678.01A.11R.1839.07 | 0.117911881 |  |  |  |  |
| TCGA.AD.6963.01A.11R.1928.07 | 0.455228 |  |  |  |  |
| TCGA.A6.5665.01B.03R.2302.07 | 0.147167814 |  |  |  |  |
| TCGA.AD.6901.01A.11R.1928.07 | -0.166774141 |  |  |  |  |
| TCGA.5M.AAT4.01A.11R.A41B.07 | 0.03396046 |  |  |  |  |
| TCGA.DM.A0XD.01A.12R.A155.07 | -0.2908179 |  |  |  |  |
| TCGA.A6.6141.01A.11R.1774.07 | 0.643551846 |  |  |  |  |
| TCGA.AA.3681.01A.01R.0905.07 | 0.47294442 |  |  |  |  |
| TCGA.A6.5666.01A.01R.1653.07 | -0.346685547 |  |  |  |  |
| TCGA.D5.6924.01A.11R.1928.07 | -0.079104934 |  |  |  |  |
| TCGA.5M.AAT6.01A.11R.A41B.07 | -0.114620933 |  |  |  |  |
| TCGA.AA.3509.01A.01R.1410.07 | 0.087973621 |  |  |  |  |
| TCGA.CK.6748.01A.11R.1839.07 | -0.585346186 |  |  |  |  |
| TCGA.AA.3525.01A.02R.0826.07 | 0.107440567 |  |  |  |  |
| TCGA.A6.A56B.01A.31R.A28H.07 | -0.001453428 |  |  |  |  |
| TCGA.AA.3972.01A.01R.1022.07 | -0.25370929 |  |  |  |  |
| TCGA.A6.2686.01A.01R.A32Z.07 | -0.336244922 |  |  |  |  |
| TCGA.G4.6311.01A.11R.1723.07 | -0.198660573 |  |  |  |  |
| TCGA.AA.3861.01A.01R.1022.07 | 0.526134526 |  |  |  |  |
| TCGA.G4.6625.01A.21R.1774.07 | 0.065423074 |  |  |  |  |
| TCGA.AD.6965.01A.11R.1928.07 | 0.618869351 |  |  |  |  |
| TCGA.AA.A00K.01A.02R.A002.07 | 0.368405858 |  |  |  |  |
| TCGA.A6.6652.01A.11R.1774.07 | 0.345272714 |  |  |  |  |
| TCGA.A6.A5ZU.01A.11R.A28H.07 | -0.207638089 |  |  |  |  |
| TCGA.AA.A00O.01A.02R.A089.07 | 0.019639276 |  |  |  |  |
| TCGA.A6.6650.01A.11R.A278.07 | 0.356523939 |  |  |  |  |
| TCGA.AA.3549.01A.02R.0821.07 | 0.339293044 |  |  |  |  |
| TCGA.AA.3970.01A.01R.1022.07 | 0.488680126 |  |  |  |  |
| TCGA.AA.3930.01A.01R.1022.07 | -0.062530584 |  |  |  |  |
| TCGA.G4.6627.01A.11R.1774.07 | 0.091483233 |  |  |  |  |
| TCGA.F4.6704.01A.11R.1839.07 | -0.666813005 |  |  |  |  |
| TCGA.CM.5349.01A.21R.1723.07 | -0.38256229 |  |  |  |  |
| TCGA.AA.3518.01A.02R.0826.07 | 0.502970819 |  |  |  |  |
| TCGA.AA.3815.01A.01R.1022.07 | 0.422484017 |  |  |  |  |
| TCGA.AA.3526.01A.02R.A32Z.07 | 0.002082852 |  |  |  |  |
| TCGA.QL.A97D.01A.12R.A41B.07 | 0.714311863 |  |  |  |  |
| TCGA.AA.3939.01A.01R.1022.07 | -0.006284494 |  |  |  |  |
| TCGA.AA.3814.01A.01R.0905.07 | -0.182659277 |  |  |  |  |
| TCGA.AA.A01V.01A.23R.A083.07 | 0.767902407 |  |  |  |  |
| TCGA.AZ.4313.01A.01R.1410.07 | -0.172146445 |  |  |  |  |
| TCGA.AA.3872.01A.01R.1022.07 | -0.312474058 |  |  |  |  |
| TCGA.A6.3810.01A.01R.A278.07 | -0.260593138 |  |  |  |  |
| TCGA.AD.6964.01A.11R.1928.07 | -0.810831933 |  |  |  |  |
| TCGA.A6.2681.01A.01R.1410.07 | -0.255523238 |  |  |  |  |
| TCGA.A6.2671.01A.01R.1410.07 | -0.207191326 |  |  |  |  |
| TCGA.CA.6719.01A.11R.1839.07 | -0.04985901 |  |  |  |  |
| TCGA.CM.4751.01A.02R.1839.07 | 0.474244196 |  |  |  |  |
| TCGA.AA.3692.01A.01R.0905.07 | 0.228945131 |  |  |  |  |
| TCGA.G4.6294.01A.11R.1774.07 | 0.2695372 |  |  |  |  |
| TCGA.D5.6540.01A.11R.1723.07 | 0.172461569 |  |  |  |  |
| TCGA.D5.6898.01A.11R.1928.07 | -0.014376041 |  |  |  |  |
| TCGA.G4.6297.01A.11R.1723.07 | -0.168231397 |  |  |  |  |
| TCGA.G4.6317.02A.11R.2066.07 | -0.595930119 |  |  |  |  |
| TCGA.AA.3821.01A.01R.1022.07 | 0.264451249 |  |  |  |  |
| TCGA.A6.2674.01A.02R.0821.07 | -1.607673773 |  |  |  |  |
| TCGA.F4.6854.01A.11R.1928.07 | 0.303412091 |  |  |  |  |
| TCGA.G4.6295.01A.11R.1723.07 | 0.736476164 |  |  |  |  |
| TCGA.A6.2684.01A.01R.1410.07 | -0.503467192 |  |  |  |  |
| TCGA.A6.6650.01A.11R.1774.07 | 0.344016823 |  |  |  |  |
| TCGA.AA.3696.01A.01R.0905.07 | 0.25353521 |  |  |  |  |
| TCGA.A6.6780.01A.11R.1839.07 | -0.051959479 |  |  |  |  |
| TCGA.AA.3866.01A.01R.1022.07 | -0.525648121 |  |  |  |  |
| TCGA.AA.3862.01A.01R.1022.07 | 0.184854696 |  |  |  |  |
| TCGA.AA.3855.01A.01R.1022.07 | 0.452356213 |  |  |  |  |
| TCGA.AA.A01Z.01A.11R.A083.07 | 0.113667475 |  |  |  |  |
| TCGA.AA.3678.01A.01R.0905.07 | 0.422744382 |  |  |  |  |
| TCGA.CK.5914.01A.11R.1653.07 | 0.060179855 |  |  |  |  |
| TCGA.NH.A8F7.06A.31R.A41B.07 | -0.585999531 |  |  |  |  |
| TCGA.CM.4748.01A.01R.1410.07 | 0.347703199 |  |  |  |  |
| TCGA.AY.6197.01A.11R.1723.07 | 0.641919549 |  |  |  |  |
| TCGA.A6.6781.01A.22R.1928.07 | -1.410052003 |  |  |  |  |
| TCGA.DM.A1D4.01A.21R.A155.07 | 0.470555204 |  |  |  |  |
| TCGA.AA.3968.01A.01R.1022.07 | 0.222500119 |  |  |  |  |
| TCGA.AA.3713.01A.21R.1723.07 | 0.528881239 |  |  |  |  |
| TCGA.CA.5797.01A.01R.1653.07 | -0.172605666 |  |  |  |  |
| TCGA.G4.6310.01A.11R.1723.07 | -0.399735885 |  |  |  |  |
| TCGA.A6.5657.01A.01R.A32Z.07 | 0.375188966 |  |  |  |  |
| TCGA.A6.5667.01A.21R.1723.07 | -0.023359833 |  |  |  |  |
| TCGA.NH.A50U.01A.33R.A37K.07 | -0.120868039 |  |  |  |  |
| TCGA.AA.A02J.01A.01R.A00A.07 | 0.061371559 |  |  |  |  |
| TCGA.A6.2682.01A.01R.1410.07 | -0.225788339 |  |  |  |  |
| TCGA.D5.6932.01A.11R.1928.07 | 0.041098655 |  |  |  |  |
| TCGA.D5.6929.01A.31R.1928.07 | -0.064683998 |  |  |  |  |
| TCGA.A6.2675.01A.02R.1723.07 | -0.401987242 |  |  |  |  |
| TCGA.A6.2684.01A.01R.A278.07 | -0.245247392 |  |  |  |  |
| TCGA.A6.6654.01A.21R.1839.07 | -1.282495943 |  |  |  |  |
| TCGA.A6.A567.01A.31R.A28H.07 | 0.127137562 |  |  |  |  |
| TCGA.AA.3666.01A.02R.0905.07 | 0.12339488 |  |  |  |  |
| TCGA.DM.A1HA.01A.11R.A155.07 | 0.125778162 |  |  |  |  |
| TCGA.CA.6717.01A.11R.1839.07 | -0.276712666 |  |  |  |  |
| TCGA.CM.6163.01A.11R.1653.07 | 0.151646994 |  |  |  |  |
| TCGA.CM.5862.01A.01R.1653.07 | -0.43374523 |  |  |  |  |
| TCGA.D5.6534.01A.21R.1928.07 | -2.063019 |  |  |  |  |
| TCGA.A6.6137.01A.11R.1774.07 | 0.59840971 |  |  |  |  |
| TCGA.AZ.6605.01A.11R.1839.07 | -0.457904525 |  |  |  |  |
| TCGA.AA.3984.01A.02R.1022.07 | 0.406731791 |  |  |  |  |
| TCGA.CA.6716.01A.11R.1839.07 | -0.024857841 |  |  |  |  |
| TCGA.AA.A01I.01A.02R.A089.07 | 0.1939441 |  |  |  |  |
| TCGA.AA.3495.01A.01R.1410.07 | 0.273564546 |  |  |  |  |
| TCGA.AA.A02F.01A.01R.A089.07 | 0.050476303 |  |  |  |  |
| TCGA.G4.6626.01A.11R.1774.07 | 0.244992798 |  |  |  |  |
| TCGA.AA.A02Y.01A.43R.A32Y.07 | 0.348775408 |  |  |  |  |
| TCGA.CM.6674.01A.11R.1839.07 | -0.042455535 |  |  |  |  |
| TCGA.A6.5656.01B.02R.A277.07 | -0.548977195 |  |  |  |  |
| TCGA.AA.3693.01A.01R.0905.07 | 0.107327421 |  |  |  |  |
| TCGA.D5.6931.01A.11R.1928.07 | 0.239197747 |  |  |  |  |
| TCGA.AA.3556.01A.01R.0821.07 | 0.125327907 |  |  |  |  |
| TCGA.AZ.4615.01A.01R.1410.07 | 0.063814591 |  |  |  |  |
| TCGA.AA.3941.01A.01R.1022.07 | 0.369207281 |  |  |  |  |
| TCGA.D5.6538.01A.11R.1723.07 | -0.047544815 |  |  |  |  |
| TCGA.AY.A71X.01A.12R.A37K.07 | 0.387252262 |  |  |  |  |
| TCGA.AA.3858.01A.01R.0905.07 | 0.001888337 |  |  |  |  |
| TCGA.AA.A02H.01A.01R.A089.07 | 0.24760691 |  |  |  |  |
| TCGA.D5.6928.01A.11R.1928.07 | -2.198427732 |  |  |  |  |
| TCGA.AA.3818.01A.01R.0905.07 | 0.313626003 |  |  |  |  |
| TCGA.AA.A01C.01A.01R.A00A.07 | 0.043667151 |  |  |  |  |
| TCGA.CM.6164.01A.11R.1653.07 | 0.279448108 |  |  |  |  |
| TCGA.AA.A00Z.01A.01R.A002.07 | 0.372902429 |  |  |  |  |
| TCGA.AA.3534.01A.01R.0821.07 | 0.377246457 |  |  |  |  |
| TCGA.AA.3672.01A.01R.0905.07 | -0.134853651 |  |  |  |  |
| TCGA.A6.2678.01A.01R.0821.07 | 0.22800944 |  |  |  |  |
| TCGA.AA.3510.01A.01R.1410.07 | 0.074585005 |  |  |  |  |
| TCGA.DM.A1D6.01A.21R.A155.07 | -0.129021645 |  |  |  |  |
| TCGA.CM.6680.01A.11R.1839.07 | 0.291358867 |  |  |  |  |
| TCGA.AA.3875.01A.01R.0905.07 | 0.228651352 |  |  |  |  |
| TCGA.CM.5348.01A.21R.1723.07 | -0.61712655 |  |  |  |  |
| TCGA.CM.6171.01A.11R.1653.07 | 0.334667605 |  |  |  |  |
| TCGA.A6.2677.01A.01R.0821.07 | 0.330140851 |  |  |  |  |
| TCGA.AZ.5407.01A.01R.1723.07 | 0.626318422 |  |  |  |  |
| TCGA.AA.3977.01A.01R.1022.07 | 0.202872735 |  |  |  |  |
| TCGA.DM.A285.01A.11R.A16W.07 | 0.043246184 |  |  |  |  |
| TCGA.AZ.6599.01A.11R.1774.07 | 0.500586177 |  |  |  |  |
| TCGA.QG.A5YX.01A.11R.A28H.07 | 0.353472532 |  |  |  |  |
| TCGA.A6.3808.01A.01R.1022.07 | -0.248867355 |  |  |  |  |
| TCGA.DM.A1DB.01A.11R.A155.07 | 0.655896965 |  |  |  |  |
| TCGA.AA.3529.01A.02R.0821.07 | 0.067867298 |  |  |  |  |
| TCGA.WS.AB45.01A.11R.A41B.07 | -3.79908708 |  |  |  |  |
| TCGA.AA.A01P.01A.21R.A083.07 | -0.253369569 |  |  |  |  |
| TCGA.AA.3502.01A.01R.1410.07 | 0.487567082 |  |  |  |  |
| TCGA.CA.5254.01A.21R.1839.07 | 0.034885485 |  |  |  |  |
| TCGA.D5.5537.01A.21R.1928.07 | 0.25155929 |  |  |  |  |
| TCGA.A6.5661.01B.05R.2302.07 | -0.80863228 |  |  |  |  |
| TCGA.QG.A5YW.01A.11R.A28H.07 | 0.456786372 |  |  |  |  |
| TCGA.NH.A5IV.01A.42R.A37K.07 | 0.357741563 |  |  |  |  |
| TCGA.A6.5661.01A.01R.1653.07 | 0.308003336 |  |  |  |  |
| TCGA.D5.6920.01A.11R.1928.07 | 0.475469207 |  |  |  |  |
| TCGA.CK.4947.01B.11R.1653.07 | 0.202715192 |  |  |  |  |
| TCGA.QG.A5YV.01A.11R.A28H.07 | 0.499411117 |  |  |  |  |
| TCGA.A6.2683.01A.01R.0821.07 | -0.330684929 |  |  |  |  |
| TCGA.AA.A024.01A.02R.A00A.07 | 0.285838563 |  |  |  |  |
| TCGA.A6.2680.01A.01R.1410.07 | 0.242182303 |  |  |  |  |
| TCGA.AZ.6603.01A.11R.1839.07 | 0.000420031 |  |  |  |  |
| TCGA.F4.6807.01A.11R.1839.07 | -0.363892147 |  |  |  |  |
| TCGA.AA.3842.01A.01R.1022.07 | -0.261526166 |  |  |  |  |
| TCGA.A6.6651.01A.21R.1839.07 | -1.088997531 |  |  |  |  |
| TCGA.AA.3667.01A.01R.0905.07 | -0.067337278 |  |  |  |  |
| TCGA.D5.6926.01A.11R.1928.07 | -0.310718004 |  |  |  |  |
| TCGA.AY.A8YK.01A.11R.A41B.07 | 0.36298764 |  |  |  |  |
| TCGA.CM.6676.01A.11R.1839.07 | 0.307658037 |  |  |  |  |
| TCGA.AD.6899.01A.11R.1928.07 | -0.124424871 |  |  |  |  |
| TCGA.AA.3986.01A.02R.1022.07 | 0.473348166 |  |  |  |  |
| TCGA.AA.A01T.01A.21R.A16W.07 | -0.08822547 |  |  |  |  |
| TCGA.A6.5656.01A.21R.1839.07 | 0.176630925 |  |  |  |  |
| TCGA.CM.6679.01A.11R.1839.07 | 0.123335116 |  |  |  |  |
| TCGA.F4.6570.01A.11R.1774.07 | -0.333694307 |  |  |  |  |
| TCGA.CM.5868.01A.01R.1653.07 | -0.09146249 |  |  |  |  |
| TCGA.CK.4951.01A.01R.1410.07 | -0.23241535 |  |  |  |  |
| TCGA.G4.6320.01A.11R.1723.07 | 0.603631756 |  |  |  |  |
| TCGA.CA.5256.01A.01R.1410.07 | -0.157936472 |  |  |  |  |
| TCGA.DM.A28E.01A.11R.A32Y.07 | 0.063292505 |  |  |  |  |
| TCGA.AA.A00Q.01A.01R.A002.07 | 0.387830291 |  |  |  |  |
| TCGA.D5.5540.01A.01R.1653.07 | -0.221964142 |  |  |  |  |
| TCGA.G4.6321.01A.11R.1723.07 | 0.319712687 |  |  |  |  |
| TCGA.G4.6588.01A.11R.1774.07 | 0.179611139 |  |  |  |  |
| TCGA.AA.3852.01A.01R.0905.07 | -0.013230413 |  |  |  |  |
| TCGA.AA.3812.01A.01R.0905.07 | -0.592621269 |  |  |  |  |
| TCGA.3L.AA1B.01A.11R.A37K.07 | 0.638214917 |  |  |  |  |
| TCGA.AA.3831.01A.01R.0905.07 | 0.349600248 |  |  |  |  |
| TCGA.DM.A282.01A.12R.A16W.07 | -0.112233945 |  |  |  |  |
| TCGA.CK.5915.01A.11R.1653.07 | 0.088343464 |  |  |  |  |
| TCGA.AA.3680.01A.01R.0905.07 | 0.736456619 |  |  |  |  |
| TCGA.AZ.4308.01A.01R.1410.07 | -0.538488154 |  |  |  |  |
| TCGA.DM.A28H.01A.11R.A16W.07 | -0.027734655 |  |  |  |  |
| TCGA.AA.3955.01A.02R.1022.07 | 0.020022833 |  |  |  |  |
| TCGA.AA.3506.01A.01R.1410.07 | 0.161045762 |  |  |  |  |
| TCGA.AZ.6600.01A.11R.1774.07 | 0.054184683 |  |  |  |  |
| TCGA.CM.4752.01A.01R.1410.07 | 0.038544285 |  |  |  |  |
| TCGA.G4.6315.01A.11R.1723.07 | 0.259530564 |  |  |  |  |
| TCGA.DM.A1D8.01A.11R.A155.07 | 0.326621555 |  |  |  |  |
| TCGA.CM.4743.01A.01R.1723.07 | -0.098010972 |  |  |  |  |
| TCGA.AD.6895.01A.11R.1928.07 | 0.440910399 |  |  |  |  |
| TCGA.AA.3679.01A.02R.0905.07 | -0.022497502 |  |  |  |  |
| TCGA.CK.6746.01A.11R.1839.07 | 0.456806919 |  |  |  |  |
| TCGA.G4.6628.01A.11R.1839.07 | 0.287202519 |  |  |  |  |
| TCGA.AA.A004.01A.01R.A00A.07 | 0.108058176 |  |  |  |  |
| TCGA.DM.A28A.01A.21R.A32Y.07 | 0.013510957 |  |  |  |  |
| TCGA.G4.6317.01A.11R.1723.07 | 0.14828634 |  |  |  |  |
| TCGA.A6.5664.01A.21R.1839.07 | -0.163784316 |  |  |  |  |
| TCGA.AA.3952.01A.01R.1022.07 | -0.421710348 |  |  |  |  |
| TCGA.AZ.6608.01A.11R.1839.07 | 0.14317841 |  |  |  |  |
| TCGA.CK.6751.01A.11R.1839.07 | 0.021760586 |  |  |  |  |
| TCGA.AD.A5EK.01A.11R.A28H.07 | 0.339697649 |  |  |  |  |
