# Supplementary material for: Infiltrating T-cell abundance combined with EMT-related gene expression as a prognostic factor of colon cancer
Source: Bioengineered. 2021 Jun 27;12(1):2688–701. doi: 10.1080/21655979.2021.1939618 (PMC8806648; doi:10.1080/21655979.2021.1939618)
Supplement: Supplemental Material [file KBIE_A_1939618_SM4068.zip › supplementary/Table_S3.docx]

| **Table S3:P value for differential expression of seven proteins.** | | | | | |
| --- | --- | --- | --- | --- | --- |
| GeneSymbol | p_value |  |  |  |  |
| TPM1 | 0.016 |  |  |  |  |
| LAMC1 | 0.197 |  |  |  |  |
| POSTN | NA |  |  |  |  |
| CCN1 | NA |  |  |  |  |
| MGP | 0.72 |  |  |  |  |
| PCOLCE2 | 0.15 |  |  |  |  |
| DPYSL3 | 0.4 |  |  |  |  |
